# The prevalence and determinants of unmet healthcare needs in Bulgaria

**DOI:** 10.1371/journal.pone.0312475

**Published:** 2024-10-29

**Authors:** Ivan Maslyankov, Mónica Hernández

**Affiliations:** School of Medicine and Population Health, University of Sheffield, Sheffield, United Kingdom; Universiti Sains Malaysia Institut Perubatan dan Pengigian Termaju, MALAYSIA

## Abstract

Self-reported unmet healthcare needs are a useful indicator of access to healthcare, but there is little research from low- and middle-income countries. This study sought to investigate the prevalence and determinants of unmet needs arising from wait times, distance/transportation and financial affordability in Bulgaria using European Health Interview Survey data. We explored associations between individual characteristics and the probability of reporting unmet need by fitting logistic regression models to the data. Unaffordability-related unmet needs were the most cited barrier to access. The largest proportion of people reported unmet dental care needs (14%) or unmet needs due to unaffordability of medicines (8%); distance/transportation problems were the least cited reason (3%). People with poor physical and mental health had a substantially higher probability of experiencing unmet needs. Those with a disability, less disposable income, less social support and lower educational attainment were also more likely to report unmet healthcare needs. People who lived in rural areas experienced specific barriers to access, most notably from distance or transportation issues. Unmet healthcare needs lead to inefficiencies for the healthcare system and are more prevalent among already disadvantaged societal groups. Ensuring better access to healthcare is therefore important from both the efficiency and equity perspectives.

## Introduction

### Universal health coverage, access and healthcare need

Ensuring access to good quality healthcare that meets the health needs of a population (universal health coverage, UHC) is a key objective of most health systems, recognised at the highest level by the European Union (EU) and reflected in the European Pillar of Social Rights [1]. Delivering UHC is a complex task that requires coordinated efforts across various sectors of a country’s health system, including strengthening the health workforce and infrastructure, ensuring financial sustainability, expanding access to services and strengthening governance and accountability, among other things. As such, access to healthcare is a key aspect of UHC and is affected by both supply-side (system) and demand-side (population) factors [2–4]. A related concept is that of (unmet) need. Unmet healthcare need has been defined as ‘*[the] instances in which people need healthcare but do not receive it because of access barriers*’ [5], which ultimately circles back to the definitions of need and access. Alternatively, a definition by Carr and Wolfe [6] states that unmet needs are ‘*the differences*, *if any*, *between those services judged necessary to deal appropriately with defined health problems and those services actually received*’. The second definition is slightly broader in that it better captures that unmet needs can also arise from delayed and sub-optimal services, besides from non-use [7]. Both definitions are quite subjective, but this is common for need theories [8]. Unmet healthcare needs can also be thought of as arising on the supply or the demand side, based on which barriers to access are experienced. System factors resulting in unmet need can be long waiting times or the cost of care; personal characteristics leading to unmet need include individual preferences and time constraints. This distinction is important from a policy perspective as the former are more easily amendable to policy, whereas the latter may reflect individuals’ preferences that are difficult to attend [9].

### Unmet healthcare needs as an indicator

There are two main approaches to investigating barriers to healthcare access. The first is to use indicators of health or service utilisation, which can be observable characteristics of the population (e.g. measures of ill-health or disease prevalence) or of the system (e.g. physician visit rates). This approach may be useful to study the supply-side barriers to access, but it does not take account of the appropriateness, acceptability and (cost-)effectiveness of care [2]. Other methodological issues have also been described [10]. An alternative is to study the (mis)alignment of supply and need, i.e. unmet healthcare needs. Though proxies for how well needs are met can be used (e.g. referral waiting times), the direct measurement of unmet need is more common. Directly measured unmet need could be either ‘*clinical’* or ‘*subjective’* [11]. Clinical unmet need is assessed by an appropriate person (usually a clinician) and is based on guidelines/best practices so it is more objective, but it is specific to a narrow set of conditions/services [11]. Subjective unmet need is self-reported so… undeniably subjective. But there are notable advantages: firstly, it has been argued that individuals are best suited to judge their health status [12], and hence the presence of unattended needs [13]. Secondly, it does not rely on respondents’ use of healthcare services. Also, self-reported data are prone to collection through surveys. Some disadvantages are that subjective unmet need indicators could capture respondents’ perceptions not based on actual need, they are prone to recall bias, and do not capture unrealised unmet need. Finally, because of methodological differences data from surveys is very difficult to compare, both between countries and between surveys [7, 14].

### The determinants and consequences of unmet healthcare needs

A number of studies have explored the relationships between unmet healthcare needs and health, socioeconomic and demographic characteristics. However, research has been conducted primarily in the context of higher-income countries, notably the United States [15–18], Canada [11, 19–21] and Europe [13, 22–27]. Some factors consistently emerge as related to unmet healthcare needs, such as (worse) self-perceived health, being female or an immigrant, having chronic conditions, being unemployed and rural residence. Studies also tend to agree on the importance of education, age, insurance status and social capital and support, albeit to a lesser extent. Studies have used different sets of explanatory variables, though it has to be noted that this is often due to limitations in the datasets. On the national level, unmet needs have been found to be higher in countries with larger income inequalities [24] and in countries where out-of-pocket (OOP) payments represent a higher proportion of total health expenditure [28].

An important question is why the presence (or lack of) unmet healthcare needs should matter to policymakers. In fact, in many cases their presence would not be a particular concern—if they are subjectively demanded, but medically unnecessary, if they are not effective unmet needs (i.e. there is no capacity to benefit), or if the care is delayed, but will be provided within reason [29]. However, in other cases the presence of unmet needs indicates shortcomings of the healthcare system, which could be amendable to policy. This relies on the notion that there are direct health consequences from the insufficient utilisation of healthcare. There is some evidence for this, showing that current unmet needs are a predictor of worse health in later years [10, 29–32], though some authors have failed to find a significant relationship [33]. Evidence exists that certain types of unmet needs may be associated with higher or lower risk of hospital admission [34, 35]. Unfortunately, the empirical literature on the health and quality of life implications of unmet healthcare needs remains scarcer than the one on the determinants of unmet needs. It does, however, show that self-reported unmet healthcare need is a valid and meaningful measure of barriers to access [10]. Beyond the efficiency perspective, targeting unmet needs is also important from the equity perspective. As previously noted, the prevalence of unmet needs tends to be higher in certain societal groups, leading to increased suffering and reduced quality of life among people who are often already disadvantaged.

### The Bulgarian healthcare system

Bulgaria’s healthcare system is based on the principles of equity, universality and sustainability and in principle provides universal coverage to all its citizens and legal residents [36, 37]. Since its reform in 1998 the system is of Bismarckian type with compulsory and voluntary health insurance contributions made by employees, employers, and the state [38, 39]. By law, all citizens and residents should be insured. However, in 2019 only 85% of the resident population was covered [40]. Among the uninsured, 50% were Bulgarian citizens who lived abroad, 25% were permanently unemployed who experienced difficulties in paying insurance contributions and 25% could afford to pay, but did not [55]. For those insured a range of primary, secondary, and tertiary level health services and goods are covered by a statutory benefits package, however, co-payments exist for many technologies and services. Others, such as most dental and long-term care services, are capped or not covered at all [41, 42]. OOP spending is therefore very high (39% of all healthcare spending in 2019) and a major challenge for the system [41, 43].

The Ministry of Health (MoH) and the National Health Insurance Fund (NHIF) are two key actors in the healthcare system, responsible respectively for national health policy and overall system functioning and for overseeing the financing and administration of the system, including the pooling of funds and purchasing of health services [40]. Both have local branches, Regional Health Inspectorates and Regional Health Insurance Funds, yet the system remains highly centralised. The system is very hospital-centric [40, 44]. Inpatient care consistently represents the largest share of public expenditure [45], though in recent years efforts have been made to increase the share spent on ambulatory and outpatient procedures. Тhe healthcare workforce is skewed towards certain specialties and towards urban centres and the (more economically developed) Southern regions of the country [46]. Last but not least, years of political instability have impeded the implementation of major reforms. The minister of health’s seat has been one of the most volatile public positions and the frequent changes of ministers (and other responsible persons) have led to an acute lack of continuity, strategic vision, political will and ultimately, results for users of the system.

## Methods

### Data and variables

Self-reported household data from wave 3 of the European Health Interview Survey (EHIS) provided by the Bulgarian National Statistical Institute (NSI) were used. The EHIS is a harmonised, cross-sectional, observational survey undertaken in all EU countries, which collects socioeconomic, demographic and health data. The survey methodology is available in a manual published by Eurostat [47]. Briefly, EHIS wave 3 collected data from October 2019 to January 2020. The survey covered all residents aged 15 years and over living in private households. Two stage sampling on a territorial principle was implemented, firstly sampling census enumeration units and then households. The sampling frame was based on a 2011 Population Census database updated with deaths and births. All individuals belonging to a selected household were invited to be interviewed. Among 10,322 people invited to participate, 7,540 responded and fully completed a face-to-face interview. Information on non-responders is available in S1 Appendix. Data checks, cleaning and editing were done by the NSI in accordance with Eurostat guidelines.

In the survey unmet healthcare needs were captured by six questions asking about unmet needs arising from long wait times, distance or transportation problems, affordability of medical care, affordability of dental care, affordability of mental healthcare and affordability of prescribed medicines. The binary answers to these questions were used as response variables in this study. They were coded as one, if respondents replied “yes” and as zero if respondents replied “no”. People who reported “no need for healthcare” were excluded. In line with the existing literature, explanatory variables were chosen based on Andersen’s behavioural model, which postulates that access to healthcare is a function of predisposing factors capturing social, economic and cultural characteristics of an individual (typically independent of health need), enabling factors which influence the logistics of care or are necessary for access, and health need factors which include health status and behaviours [48]. Variables representing predisposing factors were: gender, age, educational attainment, civil status, employment status, immigrant status, being a carer, and social capital. Enabling factors were accounted for by income, household size and residency; health need factors—by the variables self-perceived health, body mass index (BMI), presence of a chronic condition, a depressive disorder, limitations in daily activities, alcohol consumption and smoking habits.

The variables were coded as follows: gender (*male/female*), age (*15-29/30-49/50-69/70+*), educational attainment (*primary or lower/secondary/tertiary or higher*), employment status (*employed/unemployed/retired/other economically inactive*), civil status (*single/married/divorced/widowed*). We used the self-reported amount of concern people have shown in the responder’s activities (*a lot/some/not sure*, *little or none*) and the reported number of close people (*none/1-2/3-4/5 or more*) as proxies for social capital. Dummies were used for being an immigrant, a carer, presence of a chronic condition, a depressive disorder or limitations in daily activities. Income quintiles were used as numerical data for income were not available. Adjustment for household size was also performed (*1 person/2 people/3 people/4+ people*). Residence was coded as *city/town or suburb/rural area*. Finally, the remaining health variables were coded as: self-perceived health (*very good/good/fair/bad/very bad*), BMI (*underweight/normal/overweight or obese*), alcohol drinking (*regular/irregular/never*) and smoking habits (*every day/irregularly/never*). Further information on the choice of variables can be found in S2 Appendix.

### Empirical framework

To investigate the associations between the outcome and explanatory variables logit models for each component of unmet need were fitted to the data. All models were first estimated with a full set of explanatory variables. Variables were then excluded from a regression if they were grossly insignificant *and* their theoretical link to the outcome was weak. The likelihood ratio test (LRtest) and information criteria were used to decide the final models’ specification. Lastly, variables were excluded if there were concerns arising from the sample size (i.e. if there were very few observations in one or more categories).

Within the final models data were weighted at individual level (weights provided by the EHIS) to make the results representative of the general population. The Hosmer-Lemeshow test and calibration belt plots were used to assess model calibration. The link test was used to assess for model misspecification. As sensitivity analyses, the regressions were rerun after observations whose leverage was more than 5% were excluded and with estimations of simple joint models with an individual specific effect (a latent variable). Results were reported as odds ratios and conditional expected probabilities. Where appropriate, graphical presentation was also used. p<0.05 was considered statistically significant; results at p<0.1 were acknowledged. All analyses were performed on Stata 17 (StataCorp, College Station, TX, USA).

All analyses were of de-identified data. As per the authors’ institutional regulations, the use of de-identified data does not necessitate obtaining participant consent.

## Results

### Summary statistics

The original EHIS sample consisted of 7,540 people. Immigrants, underweight people and those with a depressive disorder were underrepresented (1%, 2% and 5%, respectively). Detailed characteristics of the sample including information on missing data are presented in S3 Appendix. Missing data for the explanatory variables did not exceed 5%, except for depressive disorder status (6.5%). The most cited reason for unmet needs was due to unaffordable dental care (14%), followed by unaffordability of medical care and prescribed medicines (8% each) (Table 1). Unaffordable mental healthcare and long wait times resulted in unmet needs in 5% of each corresponding sample. The least cited reason for unmet need was distance or transportation issues (3%). Among those reporting unmet needs, there were more women, older people, people with a secondary education and people in the lower income quintiles (S3 Appendix). No discernible trends were seen with regards to residence status and social capital.

**Table 1:** Prevalence of met and unmet need among people who reported need for healthcare (number and unweighted percentage).

	Wait time was too long	Distance or transport problems	Could not afford medical care	Could not afford dental care	Could not afford pre-scribed drugs	Could not afford mental healthcare
	N (%)	N (%)	N (%)	N (%)	N (%)	N (%)
**Met**	4,219 (95)	4,230 (97)	4,134 (92)	2,942 (86)	3,838 (92)	1,447 (95)
**Unmet**	202 (5)	149 (3)	342 (8)	485 (14)	316 (8)	78 (5)
**Total**	4,421 (100)	4,379 (100)	4,476 (100)	3,427 (100)	4,154 (100)	1,525 (100)

### Determinants of unmet health care needs

Due to the small number of observations immigrant status was excluded as an explanatory variable and BMI was reduced to a dummy for being overweight/obese. The six initial models were then tested with the following set of variables: gender, age, employment, education, income, household seize, civil status, carer status, concern, number of close people, residence status, self-perceived health status, presence of a depressive disorder, BMI status, presence of limitations in daily performance, presence of a chronic condition, smoking and drinking status. Additional variables were then excluded only if they were insignificant *and* a theoretical causal link had previously not been established (S2 and S4 Appendices).

For 3 models (unmet need due to long wait times, distance/transportation issues and unaffordability of mental healthcare), the reduced set of variables proved a better fit as assessed by the LRtest, AIC and BIC values (S4 Appendix). For the models assessing unmet needs due to unaffordability of medical care, dental care and prescribed medicines the full model was retained based on highly significant p-values from the LRtest and lower AIC values. In the models for dental care and prescribed medicines the BIC values were slightly lower for the reduced model, but BIC was expected to be less tolerant due to the relatively high number of parameters and thus degrees of freedom. The same two models failed the specification test and showed miscalibration based on the Hosmer-Lemeshow test and the associated calibration belt plots (S1 Fig). The remaining models did not display specification issues.

The fully adjusted ORs are presented in S5 Appendix. Two characteristics were associated with statistically significantly higher odds for each component of unmet need: the odds of reporting unmet needs were between 97% and 710% higher for people with a depressive disorder. Similarly, the odds of reporting unmet needs were between 54% and 155% higher for people who had limitations in daily activities. Two characteristics were associated with statistically significantly higher odds for 5 of the 6 components of unmet need. The odds of reporting unmet need were progressively higher the worse the self-perceived health status in all models and the ORs were significant in all models except for unmet needs due to unaffordable mental healthcare. The highest OR was for people with very bad health reporting unmet need due to unaffordable medical care (OR 9.36, 95%CI 3.76–23.32), followed by the value for the same group reporting unmet need due to long wait times (OR 7.33, 95%CI 2.60–20.67). The reference category was very good health. Low social support (expressed concern) was significantly associated with higher odds of experiencing unmet need for all reasons except due to long wait times with ORs ranging from 1.88 to 2.58. Social support (number of close people) was not significant in any of the models in which it was included. Compared with people with primary education, those with a tertiary education had lower odds of reporting unmet needs in all models, but statistically significant for models 2, 4 and 6 only (distance/transportation, dental care, mental healthcare). The odds were also lower for those with a secondary education in all but the mental healthcare model, but statistically significant in models 1 and 2 only (long wait times and distance/transportation issues).

Age was significantly associated with reporting unmet need due to unaffordable dental care. Compared with younger people (15–29 years old), people between 50 and 69 years had 147% higher odds and people above 70–202% higher odds. People between 50 and 69 had significantly lower odds of reporting unmet needs due to unaffordable mental healthcare (OR 0.24, 95%CI 0.06–0.94). Age was not significant in any of the other models. The odds of reporting unmet need were significantly lower for each subsequent income quintile in models 3 and 5 (medical care, prescribed drugs). They were also significantly lower for people in the 4^th^ and 5^th^ quintile in model 4 (dental care).

People living in towns/suburbs and in rural areas were at lower odds of reporting unmet needs due to long wait times (OR 0.50, 95%CI 0.33–0.74 and OR 0.64, 95%CI 0.44–0.95, respectively). Interestingly, compared with those living in densely populated urban areas, people living in towns/suburbs had significantly lower odds of reporting unmet needs due to unaffordable medical care, dental care and prescribed medicines, whereas rural dwellers had insignificantly higher odds. Finally, people living in rural areas had higher odds of experiencing unmet needs due to distance/transportation issues (OR 2.68, 95%CI 1.65–4.34). Being a carer was an insignificant predictor of reporting unmet needs in all but model 2 (distance/transportation issues) (OR 1.97, 95%CI 1.20–3.23). Married people had 126% higher odds of reporting unmet need due to distance/transportation issues and 38% lower odds of reporting unmet need due to unaffordable dental care. For the latter, widowed people also had lower odds (OR 0.48, 95%CI 0.29–0.80). Women had higher odds of reporting unmet needs for all components of unmet need, but they were only significant for unmet needs due to long wait times, affordability of dental care and affordability of mental healthcare and at the 10% level.

Other characteristics positively associated with reporting unmet need due to distance/transportation problems were unemployment (111% higher odds, significant at 10% only) and household size. Compared with a single-person household, people living in a two-person or a four-or-more-people household had 46% and 45% lower odds, respectively (significant at 10% only). People living in a three-person household had 62% lower odds (significant at 5%). Characteristics associated with reporting unmet need due to unaffordable mental health care were being retired (OR 0.41, 95%CI 0.15–1.12) and living in a three-person household (OR 2.29, 95%CI 0.96–5.46). Both associations were only significant at the 10% level. The odds of experiencing unmet needs in the presence of a chronic condition were only significantly higher in people reporting unmet need due to unattended mental health (OR 3.19, 95%CI 1.14–8.89). Being overweight or obese was not significantly associated with reporting any type of unmet need.

Table 2 reports the predicted probabilities of reporting unmet needs for each component of unmet need by gender and age. Women had a higher probability of reporting unmet needs for all types of unmet need except for distance/transportation problems, where the probabilities were the same; the difference for unaffordability of prescribed drugs was negligible. For the wait time, distance/transportation and unaffordable mental healthcare components the probability of reporting unmet needs seemed to fall with age, whereas for the other affordability components older age groups had higher probabilities. The probabilities for the youngest age group were associated with substantial uncertainty.

**Table 2:** Conditional expected probabilities of reporting unmet needs by gender and age group.

	Wait time was too long	Distance or transport problems	Could not afford medical care	Could not afford dental care	Could not afford pre-scribed drugs	Could not afford mental healthcare
**Gender**						
Male	3.9%	2.8%	6.7%	11.7%	6.6%	3.6%
Female	5.2%	2.8%	7.8%	13.9%	6.8%	5.3%
**Age group**						
15–29	5.7%	2.4%	6.9%	7.0%	5.8%	9.6%
30–49	6.3%	4.4%	6.4%	10.1%	8.3%	5.8%
50–69	4.6%	2.9%	6.8%	14.2%	5.8%	3.4%
70+.	3.6%	2.3%	8.8%	16.7%	7.0%	4.6%

**Fig 1:**
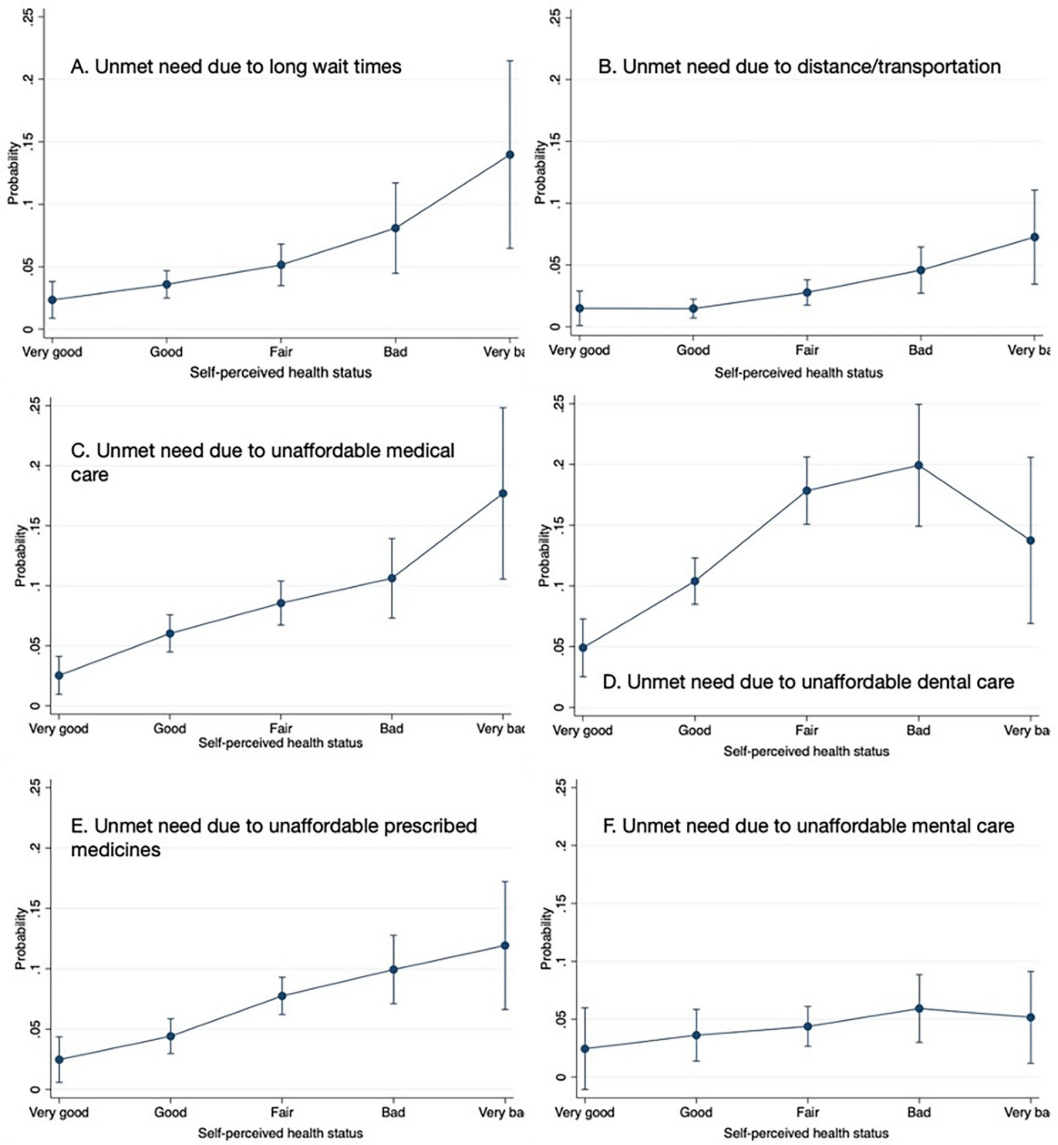
Conditional expected probabilities of reporting unmet needs by self-perceived health status.

Among people reporting unmet need due to long wait times, the probability was highest for those who were employed and lowest for the unemployed and economically inactive (S5 Appendix). Similarly, employed people had a slightly higher probability of reporting unmet needs due to unaffordable mental healthcare. In contrast, those without a permanent job had significantly higher probabilities of reporting unmet needs due to distance/transportation problems, unaffordability of medical care, dental care or prescribed drugs. The probability of experiencing all types of unmet need except for due to long wait times was higher for people who did not feel others showed concern in their activities, reaching 10.5% for experiencing unmet need due to costly medical care and 19.4% due to unaffordable dental care (S5 Appendix). A clear relationship between the probability of reporting unmet needs and self-perceived health status was observed (Fig 1). People perceiving their health as very bad had the highest probability for all types of unmet need except for unmet need due to unaffordable dental and mental healthcare, in which cases the probability was highest for people with bad health (though these differences were not always statistically significant due to large standard errors). The probabilities of experiencing all types of unmet need were up to twice higher in people who reported limitations in daily activities compared with those without, ranging from 3.5% for unmet need due to distance/transportation issues to 15.8% for unmet need due to overly costly dental care.

**Fig 2:**
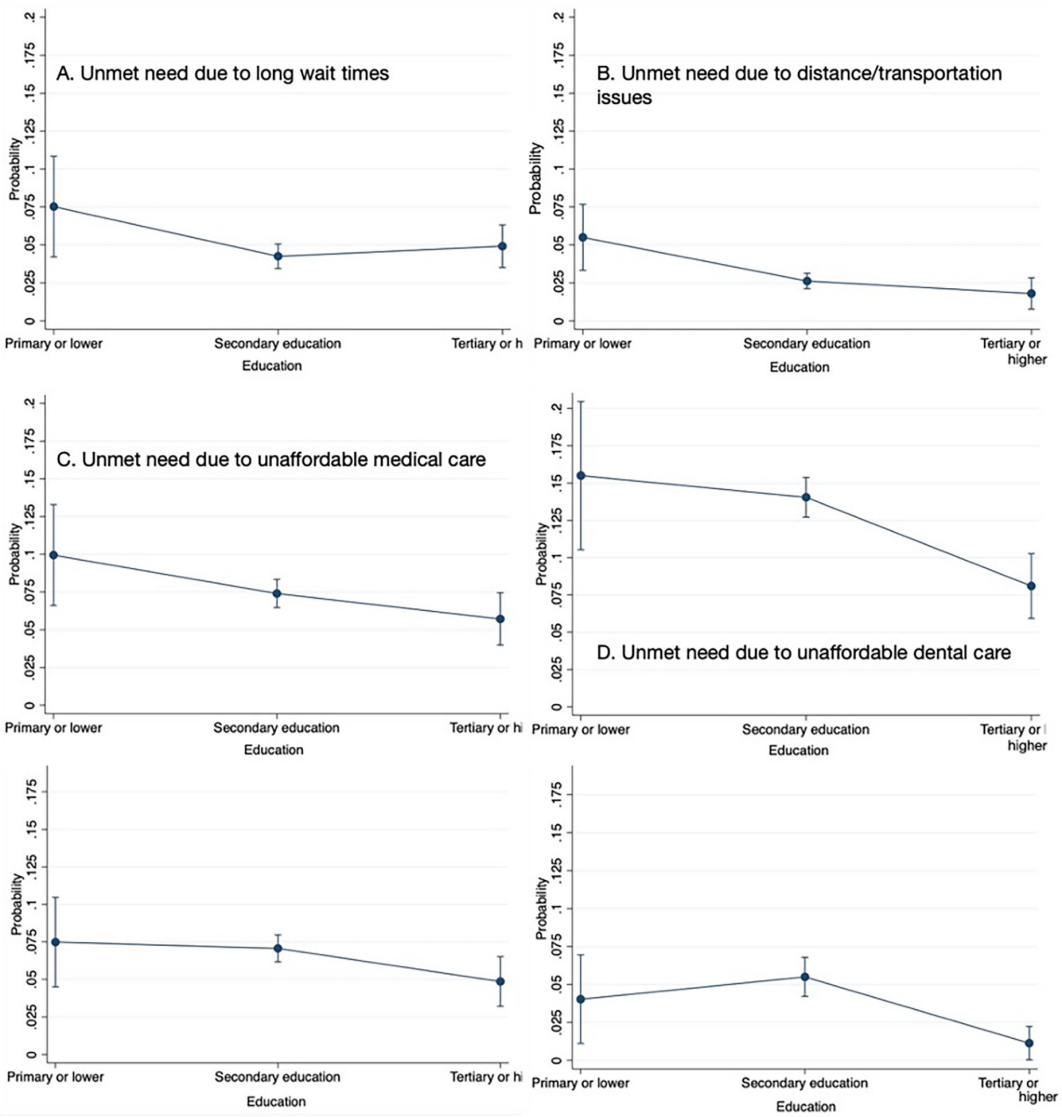
Conditional expected probabilities of reporting unmet needs by educational attainment.

The probability of reporting all types of unmet needs was much higher in people with a depressive disorder. It reached 11.8%, 22.4% and 10.9% for unmet needs due to unaffordable medical care, dental care and prescribed medicines, respectively. However, the biggest relative difference was between the probabilities of reporting unmet need due to unaffordable mental healthcare: 14.5% compared with 2.5% for people with and without a depressive disorder, respectively. Consistent with the insignificant ORs, the probabilities for people with and without a chronic condition were typically not higher in any of the groups (S5 Appendix). Generally, the likelihood of reporting unmet need was lower the more educated a person was, but the relative effect size was not big (Fig 2). However, the level of educational attainment slightly attenuated the effect of income, i.e. the probabilities of reporting unmet need for different income quintiles converged for people with a tertiary education (S2 Fig).

**Fig 3:**
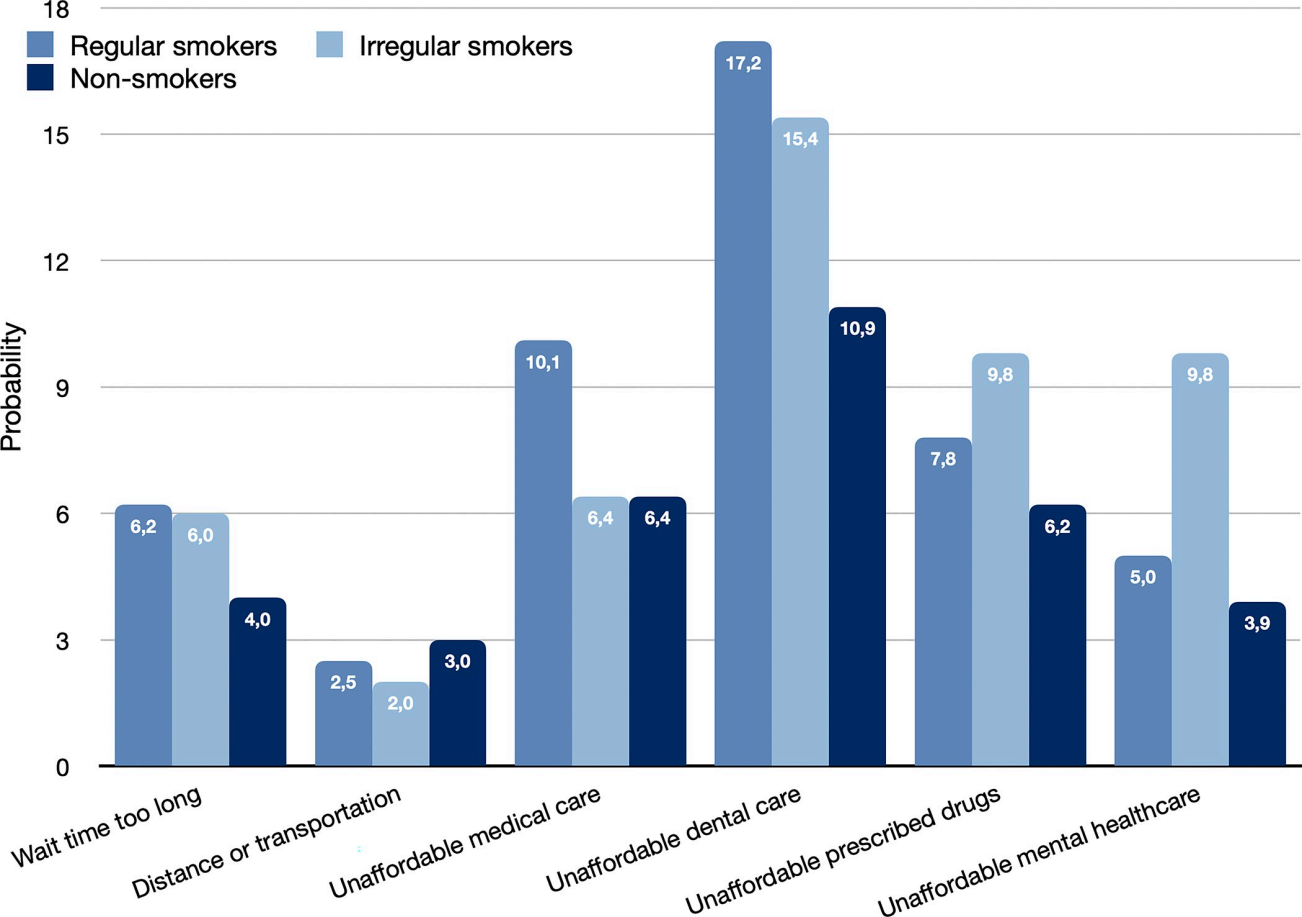
Probabilities of reporting unmet need for each component by smoking status.

**Fig 4:**
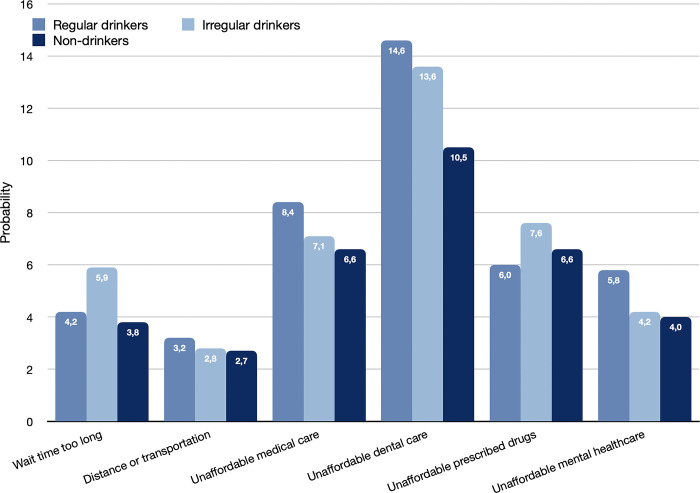
Probabilities of reporting unmet need for each component by drinking status.

Education (with gender and presence of a depressive disorder) were the only three characteristics whose effect disappeared in the first sensitivity analysis. This occurred only in the model for unmet mental healthcare needs. Finally, the likelihood of reporting unmet needs was also dependent on health behaviours such as smoking and alcohol consumption (Figs 3 and 4). For 5 out of 6 types of unmet need the probability of reporting unmet needs was lower for non-smokers than for regular smokers. In the case of unmet need due to distance/transportation issues the probability was highest for non-smokers though the absolute difference was within 1%. Similarly, non-drinkers had a lower probability than regular and irregular drinkers for all but one type of unmet need (prescribed drugs), where the difference was minor. The most notable difference was observed for unmet need due to unaffordable dental care, where non-consumption of cigarettes and alcohol reduced the probabilities of unmet need by more than 3%.

In the second sensitivity analysis, the individual effect variable was highly significant in all six models. However, overall, the results were very consistent with those from the general models in terms of direction of effect and only had singular differences in terms of the significance of the results. Most of the differences involved variables changing significance from 5% to 10% or vice versa. The results as coefficients are presented in S6 Appendix. Posterior predictions of the latent variable indicated that there was both discrete and continuous heterogeneity between the individuals (S3 Fig).

## Discussion

### Main findings

Unaffordability of medical care, dental care and prescribed medicines were the most commonly cited barrier to access, which was expected given the high prevalence of OOP payments in Bulgaria. OOP spending is used precisely for services not covered by the benefits package (such as most dental services) or for those only partially covered and therefore subject to co-payments (such as many medicines). Long wait times and distance and transportation issues represented the least cited barriers to access.

The results from the statistical analyses showed that unmet healthcare needs are unequally distributed among societal groups and it is often the already vulnerable who are more likely to experience them. Poorer self-perceived health increased the likelihood of experiencing every type of unmet need, a finding consistently reported in the literature from other countries [13, 19, 21, 23, 25, 49, 50]. This could be attributed to the fact that people in worse health have more contacts with the system and potentially also higher expectations, but the exact causal mechanisms need further investigation. Having a depressive disorder was another highly significant determinant of unmet healthcare needs, as was the presence of limitations in daily activities, but not the presence of a chronic condition. Herr and colleagues [49] and Fjær and colleagues [23] have previously found depression to be associated with unmet needs. Cavalieri has reported associations between unmet needs and the presence of a chronic condition and of limitation in daily activities [13], as have Moran and colleagues [25], but of unmet needs due to long wait times only. Limitations in daily activities and the presence of a chronic condition can be seen as proxies for disability. Two studies which have used EHIS data and a variable derived by combining the two have also found higher odds of reporting all 6 types of unmet need among disabled people [51, 52].

The effects of socioeconomic characteristics were less prominent. Educational attainment was associated with less unmet need, particularly for people with a tertiary education. However, the effect size was not very large and appeared to be related to the effect of income. Some researchers have failed to find a relationship between education and unmet needs [23], while others have found that higher education is associated with more unmet needs [21]. Therefore, our findings should be interpreted with caution. The fact that income was not associated with unmet needs due to long wait times or due to distance/transportation problems was unexpected. A possible explanation is that the income quintile was not a suitable proxy for disposable income, even after adjustment for household size. Alternatively, income may simply not be a determinant of unmet need arising from long wait times or from distance/transportation problems, which is less probable. Given that income was associated with three of the four affordability components, the lack of association between income and unmet need due to unaffordable mental healthcare can be attributed to the statistical uncertainty stemming from the smaller sample size.

With regards to age a clear and statistically significant gradient was observed only for unmet needs for dental care, presumably because the amount of dental care needed and hence, the cumulative costs increase with age, while very little dental care is covered in the benefits package. The oldest age group (70+) had a slightly higher probability of reporting unmet needs due to unaffordable medical care, likely for the same reason. It is also possible that at this stage of life there are less treatment options, which would lead to perceived unmet need. The youngest age group (15–29) had the highest probability of reporting unmet needs due to unaffordable mental healthcare. One potential explanation might be that younger people are disproportionally more aware of mental health and inherently more willing to spend on mental healthcare or simply holding higher expectations of what mental healthcare should entail. The probability of reporting unmet needs due to long wait times or distance/transportation issues was only slightly higher in the 30-49-year-olds. This age group likely reflected economically-active people, who would have less free time and would therefore perceive long waits or travels as more significant barriers. This was weakly supported by the finding that employed people also had a higher probability of unmet needs due to long wait times. However, both the age and employment effects with regards to distance/transportation-related unmet need were subject to uncertainty.

On par with previous findings [9, 13, 19, 21, 23, 50], female gender was associated with a higher probability of reporting unmet needs. However, the results were at best borderline significant and the effect size was small. Previously the higher prevalence of unmet needs among women has been attributed to attitudes to care seeking and gender norms [50]. Being a carer was only significantly associated with unmet need due to issues with distance/transportation. Since in Bulgaria the majority of carers are women, it is possible that the carer effect was captured by gender. However, social support, as proxied by the expressed concern felt by the respondents, emerged as an important factor related to unmet needs. This effect of social support on mediating access to healthcare is likely through effects on the community or family level and warrants further investigation. Social support may act as a safety net through reassurance, but also by providing access to additional income. Yet, the number of close people was an insignificant predictor in all of the models in which it was included. Further research should establish the most appropriate ways to measure social support.

People living in towns and rural areas were less likely to face long wait times, possibly due to organisational flexibility in those areas. However, living in a rural area was a highly significant predictor of unmet needs due to distance/transportation issues. This can be explained on the one hand by the prominent lack of medical personnel in most of Bulgaria’s rural areas. On the other hand, this might be related to a lack of transportation options in remote areas. Interestingly, people living in towns and suburbs were significantly less likely to face affordability barriers than those living in cities. This could be indicating a favourable disposable income to cost of care ratio, but further investigations of these results are warranted. Unemployed people were more likely to face unmet needs due to unaffordable care or medicines or due to distance/transportation problems. This is likely due to a residual income effect, but a small proportion of the effect could be attributed to the fact that in Bulgaria private insurance is almost exclusively provided as a benefit through employment.

Finally, being overweight or obese was not associated with any type of unmet need. However, smoking and alcohol consumption had a clear adverse effect. Both have well-established negative effects on health. Regular smokers and drinkers are at an increased risk of many diseases and are more likely to have healthcare needs. Additionally, the prevalence of smoking and drinking is higher in lower socioeconomic strata, where spending on alcohol and cigarettes could represent a significant proportion of disposable income.

### Strengths and limitations

Several limitations have to be noted. Due to the cross-sectional nature of the data no causal inferences as to the reasons for the observed inequities can be made. The wording of the questions on unmet need in the survey makes it hard to disentangle if unmet needs have arisen from non-use, delayed use or suboptimal use. Such information would be very informative for policy purposes and calls have been made to adjust the respective questions [7, 25]. Also, it has to be noted that data were collected up to January 2020, so any impacts of the COVID pandemic will not have been captured (the first proven case in Bulgaria was on 08 March 2020). The pandemic saw substantial reallocation and reprioritisation efforts and the prevalence of unmet needs spiked (at least temporarily) [53]. However, a strength of this study is that by using data from the EHIS, analyses of six different components of unmet need were possible. It therefore adds to the particularly understudied areas of unmet need for dental and mental healthcare in Bulgaria.

Some limitations arise from the variables used. With regards to the dependent variables, concerns persist due to the subjective nature of self-reported data. Furthermore, the components of unmet need collected through the EHIS are more concerned with the supply-side causes of unmet need. Therefore, questions on the prevalence of unmet needs due to for example fear of treatment or due to dislike of the medical personnel remain unanswered. In line with previous research using EHIS data, this study investigated unmet healthcare needs among people who reported healthcare needs only. With regards to the covariates, although the best efforts were made to include all relevant explanatory variables, data were absent for some important characteristics, such as residence (geographical region), insurance status and ethnicity. Evidence suggests that these characteristics are associated with unmet healthcare needs [13, 17, 25, 41, 54, 55]. The exclusion of variables such as immigrant status is also a limitation. The lack of continuous data and thus the inability to investigate interactions and alternative specifications of the variables is noteworthy. The omission of important variables or of interaction terms could have resulted in the misspecification observed in some of the models. Despite these limitations, this study benefitted from a rich set of explanatory variables, which allowed for the investigation of characteristics not usually accounted for in previous research. Additionally, sensitivity analyses showed that the results are robust and valid.

### Implications for policy and research

The Bulgarian health system is characterised by a high level of OOP payments. This study reconfirms that financial protection should be a top priority for policymakers, especially with regards to spending on prescribed medicines and dental care. Addressing the burden of co-payments for medicines and services has been on the policy agenda of recent governments, however, effective steps are yet to be taken. High-risk groups such as those on low incomes or in poor health should be prioritised. Unfortunately, extensions to the dental care benefits package have not been on the policy agenda. However, this should be considered given the high prevalence of unmet dental care needs identified in this study. Besides the aforementioned, policies aimed at increasing population insurance coverage are also needed. Wait times and distance/transportation issues seem to not be particular concerns for the Bulgarian healthcare system, but further investigation is needed since important disparities may have been concealed by the lack of data on geographical residence in the EHIS sample. More granular research could also investigate if wait times for specific services are problematic.

The findings regarding unmet mental healthcare needs are particularly worrying. As previously noted, it is possible that a significant proportion of unmet mental healthcare need is not realised, resulting in the low prevalence in the general population. In addition, having a depressive disorder emerged as an important determinant of all types of unmet need (including affordability-related). The systematic underfunding of mental care services is a big problem in the country. However, simply increasing funding is unlikely to bring the desired results. A complete reformation of the sector with a reassessment of priorities and a strategic outlook is needed. Reforms such as the valuation of medical labour and incentives for medical specialties related to mental healthcare have been discussed and may be appropriate supply-side measures. However, population-wide approaches based on education and health promotion can be much more impactful. The WHO has outlined three paths to transforming mental healthcare [56]: *1*. *Deepen the value and commitment we give to mental health; 2*. *Reshape environments that influence mental health*, *including homes*, *communities*, *schools*, *workplaces*, *health care services*, *natural environments;* and *3*. *Strengthen mental health care by changing where*, *how*, *and by whom mental health care is delivered and received*. Achieving meaningful results will undoubtedly require significant funding, political and social capital, but digital innovations have opened a new frontier of opportunities.

Policymakers should be aware of the associations between unmet needs and characteristics of societal groups which are already disadvantaged and vulnerable—people with disability or a depressive disorder, the less educated and the unemployed, informal carers and those without social support. Policies that effectively target these groups are likely to be multilateral and involving stakeholders from the areas of health, education, social care and beyond.

The findings of this study are an important addition to a wide gap in the literature on unmet healthcare needs in Bulgaria. However, they would greatly benefit from future research to validate the trends observed. This can include both qualitative research and quantitative analyses of newer or alternative survey data.

## Conclusion

This study was the first quantitative analysis of unmet healthcare needs in Bulgaria. The prevalence of unaffordability-related unmet needs was high, while long wait times and distance or transportation issues were not perceived as barriers to access. While the nature of the data does not support causal inferences, the findings from the statistical analyses suggest that poor physical and mental health, disability, lower income and social support and unhealthy behaviours are important determinants of unmet need. Gender and education were also associated with unmet healthcare needs. Age and residence status were determinants of specific types of unmet need only. The results indicate that in many cases it is already vulnerable societal groups that are most likely to experience unmet healthcare needs.

## Supporting information

S1 Appendix Information on non-responders in EHIS. (PDF)

S2 Appendix Information on the choice of variables in the statistical models. (PDF)

S3 Appendix Characteristics of the EHIS sample. (PDF)

S4 Appendix Variables in the statistical models. (PDF)

S5 Appendix Fully-adjusted odds ratios and conditional expected probabilities of reporting unmet needs for selected parameters. (PDF)

S6 Appendix Fully adjusted coefficients with a latent variable in each model. (PDF)

S1 Fig Calibration belt plots for each of the final regression models. (PNG)

S2 Fig Conditional expected probability of reporting unmet needs disaggregated by education and income quintile. (PNG)

S3 Fig Posterior prediction for the latent variable. (JPG)
