# Supplementary material for: The prevalence and determinants of unmet healthcare needs in Bulgaria
Source: PLoS One. 2024 Oct 29;19(10):e0312475. doi: 10.1371/journal.pone.0312475 (PMC11521248; doi:10.1371/journal.pone.0312475)
Supplement: S1 Appendix — (PDF) [file pone.0312475.s001.pdf]

S1 Appendix. Information on non-responders in EHIS

The non-response rate in other household surveys conducted in Bulgaria (SILC, LFS, AES etc.) as well as in EHIS wave 2 was taken into account when determining the sample size for EHIS wave 3. Non-response adjustment was used for the calculation of weighting factors. Non-response rate in EHIS wave 3 - 27%.

S1 Table. Summary of study participation and non-participation; EHIS wave 3 (2019)

	N
**Eligible sample cases**	10,322
**Response cases**	7,540
**Non-response cases**	2,782
Non-contact	1,736
Refusal	1,035
Inability to respond	11
Rejected interview (poor quality)	0

S2 Table. Missing observations for the variables included in the analysis, EHIS wave 3 (2019)

Missing	Gender	Age	Edu-cation	Employ-ment	Married	Immigrant	Income	Urban	Concern	Carer	BMI	Health status
N	0	0	0	4	17	0	277	0	4	319	362	309
%	0	0	0	0.05	0.23	0	3.67	0	<1	4.23	4.80	4.1

Missing	Depression	Smoker	Limited performance	Drinker	Close people
N	492	323	4	331	0
%	6.53	4.28	0.05	4.39	0

S3 Table. Income and gender characteristics of respondents with missing unmet need data

Reason		1st Q	2nd Q	3rd Q	4th Q	5th Q	Mis.	Total	Male	Female	Total
**Wait time**	No	15.66	18.37	20.81	22.05	20.32	2.80	100	42.50	57.50	100
	Yes	21.40	14.85	21.40	16.59	20.96	4.80	100	38.43	61.57	100
	Mis.	13.91	16.52	22.26	21.48	20.83	4.99	100	52.55	47.45	100
**Distance**	No	15.32	18.21	20.88	22.07	20.64	2.89	100	42.22	57.78	100
	Yes	35.37	20.73	27.44	7.32	6.10	3.05	100	40.85	59.15	100
	Mis.	13.81	16.38	21.79	21.86	21.20	4.96	100	52.62	47.38	100
**Affordability: medical**	No	14.75	18.01	20.59	22.74	20.93	2.96	100	41.57	58.43	100
	Yes	32.11	21.15	21.93	9.40	11.75	3.66	100	39.95	60.05	100
	Mis.	13.51	16.36	22.53	21.66	21.11	4.82	100	54.31	45.69	100

**Affordability: dental**	No	12.72	15.60	20.04	23.43	25.32	2.88	100	42.46	57.54	100
	Yes	24.03	24.77	22.00	14.42	11.09	3.70	100	42.51	57.49	100
	Mis.	15.94	18.16	22.35	21.26	17.98	4.31	100	49.57	50.43	100
**Affordability: drugs**	No	15.49	18.96	21.08	21.62	19.88	2.98	100	41.55	58.45	100
	Yes	33.61	24.24	20.39	10.19	7.99	3.58	100	38.29	61.71	100
	Mis.	12.61	14.94	21.86	23.10	22.87	4.61	100	53.12	46.88	100
**Affordability: mental healthcare**	No	16.96	20.10	22.35	18.24	19.46	2.89	100	40.91	59.09	100
	Yes	29.47	20.00	27.37	14.74	7.37	1.05	100	34.74	65.26	100
	Mis.	14.49	16.86	21.01	22.69	21.03	3.92	100	47.67	52.33	100

Proportions of individuals answering yes, no or with missing information on unmet need; people stating no need for healthcare included in the missing category. Mis. = missing, Total = row total, Q = income quintile

The income and gender profile of those with missing unmet need data highly resembled the profile of those who did not experience unmet need. People missing data on unmet were slightly more often employed, unemployed or otherwise inactive and less often retired (data not presented). Younger age groups were slightly overrepresented among those with missing data on unmet need (data not presented).
