# Supplementary material for: The prevalence and determinants of unmet healthcare needs in Bulgaria
Source: PLoS One. 2024 Oct 29;19(10):e0312475. doi: 10.1371/journal.pone.0312475 (PMC11521248; doi:10.1371/journal.pone.0312475)
Supplement: S2 Appendix — (PDF) [file pone.0312475.s002.pdf]

S2 Appendix. Information on the choice of variables in the statistical models

A central problem is that although Andersen's behavioural model provides guidance on which characteristics may be important, causal mechanisms have not been fully established. For some little doubt exists: income is not a perfect, but a reasonable proxy for wealth or disposable income, hence an important factor in the context of the high OOP expenditure in Bulgaria and therefore all affordability components of unmet need. Distance and transportation issues can also be directly related to the inability to pay for transport. With regards to wait times, higher income can be seen as an enabler of treatment in the private sector as well as a source for informal payments. The dataset contained only the income quintile in which the family of the respondent fell. However, the number of family members could have a substantial effect on the per capita disposable income. Equivalence scales are commonly used to adjust for this. Unfortunately, numerical data for income were not available, so as a next-best option household size was adjusted for as a separate variable. Data on population density were used in order to reflect the unequal medical personnel density, which could be a cause of unmet need, especially due to long wait times or distance/transportation issues.

Women generally face more barriers to access, attributed to complex sociocultural but also biomedical reasons[1,2]. Men on the other hand are prone to more risky and unhealthy behaviours and internalising problems and have a markedly shorter life expectancy. In Bulgaria the life expectancy gap is more than 7 years. Age is another important factor. On the one hand, unmet needs might be more prevalent among younger people, as they tend to have higher expectations and might be less experienced with navigating the healthcare system. On the other hand, older age is associated with comorbidity and polypharmacy, which may limit intervention/treatment if contraindications exist. Some other services might have explicit age-related restrictions based on marketing authorisations, cost-effectiveness or budget impact. However, in other cases older age may lead to prioritisation (as was the case with COVID vaccination). People under 18 might also experience age-related barriers as the marketing authorisations of many medicines cover only people over 18, however, off-label use is not infrequent. Another important factor is educational attainment. More educate people might be better at navigating the system, better at recognising needs and more aware of their rights as consumers of healthcare.

Characteristics of the healthcare system such as better financial protection of the retired and students and the almost exclusive provision of private health insurance through employers provided reason for the use of employment status as an explanatory variable. Also, economically inactive people were hypothesised to have more free time and so a different perception of what constitutes a long wait. Immigrants have been shown to often have worse knowledge of the healthcare system and to suffer from language barriers, though those effects may be captured by educational differences or other factors. Similarly, civil status has very often been included as an explanatory variable, even though potential causal mechanisms may already be captured by income and social support. Social capital has been shown to strongly influence behaviour in a wide range of decisions, including related to accessing healthcare, through membership in social networks or through features of social organisation, such as trust and norms[3]. However, there is no agreement on how it

---

[1] Sen, G., & Östlin, P. (2008). Gender inequity in health: why it exists and how we can change it. *Global Public Health, 3*(sup1), 1–12. doi:10.1080/17441690801900795

[2] Sorensen, G. (2000). Introduction to social determinants of health. *Women & Health,* 523–527. doi.org/10.1016/B978-012288145-9/50048-6.

[3] Quintal, C., Lourenço, Ó., Ramos, L. M., & Antunes, M. (2019). No unmet needs without needs! Assessing the role of social capital using data from European social survey 2014. *Health policy, 123*(8), 747–755. doi.org/10.1016/j.healthpol.2019.06.001

should be measured[4]. We used the self-reported amount of concern people have shown in the responder's activities and the reported number of close people as proxies for social capital, with the expectation that the probability of reporting unmet needs will be inversely related to social capital. Carers were hypothesised to have less free time and to be more likely to delay care if it is not readily accessible. Accordingly, carers were expected to be more likely to report unmet needs due to long wait times and distance/transportation issues, but not due to affordability of care or medicines.

Health variables were included based on the assumption that worse health is directly related to more healthcare needs and so more frequent contact with the system, which would increase the likelihood of experiencing unmet needs. Self-perceived health status is a very good proxy for overall health and was considered the most important health-related explanatory variable. Five categories were used: very good, good, fair, bad and very bad. BMI was conceptualised as a biomedical indicator of health status. Continuous BMI data were available, but categorisation was done in order to mitigate issues of measurement error. BMI below 18.5 was categorised as underweight and BMI above 25.0 as overweight or obese (standard cut-off values). The sample size for obesity was too small to enable meaningful analysis as a separate category. People with a chronic condition, depressive disorder or limitations in daily activities were thought to represent societal groups with particularly high needs. It was also hypothesised that they may face additional barriers to access. Disabled people may need to pay informally for special transport or consultations with priority and are more likely to already be on (potentially costly) prescribed medicines. In Bulgaria, people with a depressive disorder face limited availability of mental health care professionals and services. Population-wide lack of awareness could be another reason for delays in seeking mental healthcare. The presence of a chronic condition, depressive disorder or limitations in daily activities were specified via dummy variables. A person was categorised as having a depressive disorder if they had a score of 10 or greater on the Personal Health Questionnaire (PHQ-8) Depression Scale[5]. Finally, alcohol consumption and smoking habits were used to account for health-related behaviours. Smokers have been shown to consume excess medical care and in particular inpatient care, which has been interpreted as evidence that they are less concerned with their health[6]. Spending on tobacco and alcohol may represent a significant portion of the disposable income of poorer people. Following Moran and colleagues[7] alcohol consumption was categorised as regular, irregular or never and smoking habits as every day, irregularly or never. All of the variables listed can be thought of as social determinants of health. Consequently, they could be affecting health and access to healthcare via more complex, intersectional pathways that include realised or unrealised stigma, discrimination and stress.

---

[4] Derose, K. P., & Varda, D. M. (2009). Social capital and health care access: a systematic review. *Medical care research and review, 66*(3), 272–306. doi.org/10.1177/1077558708330428

[5] Kroenke, K., Strine, T. W., Spitzer, R. L., Williams, J. B., Berry, J. T., & Mokdad, A. H. (2009). The PHQ-8 as a measure of current depression in the general population. *Journal of affective disorders, 114*(1-3), 163–173. doi.org/10.1016/j.jad.2008.06.026

[6] Izumi, Y., Tsuji, I., Ohkubo, T., Kuwahara, A., Nishino, Y., & Hisamichi, S. (2001). Impact of smoking habit on medical care use and its costs: a prospective observation of National Health Insurance beneficiaries in Japan. *International journal of epidemiology, 30*(3), 616–623. doi.org/10.1093/ije/30.3.616

[7] Moran, V., Suhrcke, M., Ruiz-Castell, M., Barré, J., & Huiart, L. (2021). Investigating unmet need for healthcare using the European Health Interview Survey: a cross-sectional survey study of Luxembourg. *BMJ open, 11*(8), e048860. doi.org/10.1136/bmjopen-2021-048860
