# Supplementary material for: The prevalence and determinants of unmet healthcare needs in Bulgaria
Source: PLoS One. 2024 Oct 29;19(10):e0312475. doi: 10.1371/journal.pone.0312475 (PMC11521248; doi:10.1371/journal.pone.0312475)
Supplement: S3 Appendix — (PDF) [file pone.0312475.s003.pdf]

S3 Appendix. Characteristics of the EHIS sample

	N (%)
**Gender**	
Male	3,477 (46)
Female	4,063 (54)
**Age**	
15-19	342 (5)
20-24	340 (5)
25-29	356 (5)
30-34	390 (5)
35-39	450 (6)
40-44	591 (8)
45-49	694 (9)
50-54	590 (8)
55-59	595 (8)
60-64	685 (9)
65-69	709 (9)
70-74	685 (9)
75-79	524 (7)
80-84	325 (4)
85 and over	264 (3)
**Education**	
Primary or lower	474 (6)
Secondary	5,465 (73)
Tertiary or higher	1,610 (21)
**Settlement status**	
Cities	3,266 (43)
Towns and suburbs	2,485 (33)
Rural areas	1,789 (24)
**Carer**	
Yes	970 (13)
No	6,251 (87)
**BMI status**	
Underweight	151 (2)
Normal weight	2,991 (42)
Overweight and obese	4,036 (56)

	N (%)
**Employment status**	
Employed	3,405 (45)
Unemployed	649 (9)
Retired	2,695 (36)
Other economically inactive	787 (10)
**Marital status**	
Single	1,945 (26)
Married	3,847 (51)
Widowed	1,247 (17)
Divorced	484 (6)
**Immigration status**	
Not an immigrant	7,499 (>99)
Immigrant	41 (<1)
**Income**	
1st quintile	1,145 (16)
2nd quintile	1,325 (18)
3rd quintile	1,611 (22)
4th quintile	1,634 (23)
5th quintile	1,548 (21)
**Household size**	
1 person	1,228 (16)
2 people	2,286 (30)
3 people	1,536 (20)
4+ people	2,490 (33)
**Concern***	
A lot	2,808 (39)
A little	3,107 (43)
Not sure/little/none	1,290 (18)
**Depressive disorder**	
Yes	381 (5)
No	6,667 (95)
**Chronic illness**	
Yes	3,496 (46)
No	4,038 (54)

Table continued…	**N (%)**
**Self-assessed health status**	
Very good	1,682 (23)
Good	2,905 (40)
Fair	1,790 (25)
Bad	677 (9)
Very bad	177 (2)
**Close people**	
None	147 (2)
1 to 2 people	3,407 (47)
3 to 4 people	2,900 (40)
5 or more people	765 (11)
**Smoking habits**	
Smoker, every day	1,955 (27)
Smoker, irregular	472 (7)
Never	4,790 (66)
**Alcohol drinking**	
Regularly	2,388 (33)
Irregularly	2,527 (35)
Never	2,294 (32)
**Limited performance**	
Yes	1,870 (25)
No	5,666 (75)

N refers to number of respondents in the sample and % is the unweighted proportion.
**Concern is a variable representing how much concern people show in the respondent's activities. Abbreviations: BMI Body Mass Index*

	Wait time was too long	Distance or transport problems	Could not afford medical care	Could not afford dental care	Could not afford pre-scribed drugs	Could not afford mental healthcare
	N (%)	N (%)	N (%)	N (%)	N (%)	N (%)
**Gender**						
Male	76 (2)	59 (1)	137 (3)	208 (6)	126 (3)	28 (2)
Female	126 (3)	90 (2)	205 (4)	277 (7)	190 (4)	50 (3)
**Age**						
15-29	8 (<1)	2 (<1)	13 (<1)	15 (1)	7 (<1)	4 (<1)
30-49	44 (1)	21 (1)	58 (2)	89 (3)	49 (1)	14 (1)
50-69	77 (2)	52 (1)	122 (3)	208 (5)	105 (2)	29 (2)
70+	73 (1)	64 (1)	149 (3)	173 (4)	155 (3)	31 (2)
**Education**						
Primary	27 (<1)	38 (1)	52 (1)	52 (1)	41 (1)	10 (<1)
Secondary	123 (3)	101 (2)	247 (5)	381 (10)	241 (5)	64 (4)
Tertiary	52 (1)	10 (<1)	43 (1)	52 (2)	34 (1)	4 (<1)
**Marital status**						
Single	32 (1)	13 (<1)	57 (2)	86 (3)	47 (1)	13 (1)
Married	108 (2)	79 (2)	163 (3)	251 (6)	152 (3)	40 (2)
Widowed	47 (1)	50 (1)	98 (2)	109 (2)	99 (2)	18 (1)
Divorced	15 (<1)	7 (<1)	24 (1)	39 (1)	18 (<1)	7 (<1)
**Immigrant status**						
No	202 (5)	149 (3)	340 (7)	482 (13)	313 (7)	77 (4)
Yes	0 (0)	0 (0)	2 (<1)	3 (<1)	3 (<1)	1 (<1)
**Employment**						
Employed	76 (2)	22 (1)	89 (2)	149 (5)	66 (2)	20 (1)
Unemployed	9 (<1)	16 (<1)	41 (1)	55 (2)	29 (1)	7 (<1)
Retired	102 (2)	96 (2)	185 (3)	245 (6)	196 (4)	38 (2)
Other	15 (<1)	15 (<1)	27 (1)	36 (1)	25 (1)	13 (1)
**Carer status**						
No	170 (4)	123 (2)	290 (6)	407 (11)	275 (6)	66 (4)
Yes	32 (1)	26 (1)	52 (1)	78 (2)	41 (1)	12 (1)
**Concern**						
A lot	83 (2)	35 (1)	99 (2)	128 (4)	74 (2)	19 (1)
Some	80 (2)	66 (1)	144 (3)	221 (6)	153 (3)	31 (2)
Not sure/0	39 (1)	47 (1)	98 (2)	135 (4)	87 (2)	28 (2)
**Depressive disorder**						
No	153 (4)	98 (2)	262 (6)	399 (11)	238 (5)	32 (2)
Yes	49 (1)	51 (1)	80 (1)	86 (2)	78 (2)	46 (2)

N refers to number of respondents and % is the weighted percentage

Table continued…

	N (%)	N (%)	N (%)	N (%)	N (%)	N (%)
**Household size**						
1 person	52 (1)	47 (1)	98 (2)	111 (3)	99 (2)	20 (1)
2 people	66 (1)	49 (1)	103 (2)	164 (4)	95 (2)	25 (1)
3 people	34 (1)	15 (0)	57 (1)	78 (2)	49 (1)	16 (1)
4+ people	50 (1)	38 (1)	84 (2)	132 (4)	73 (2)	17 (1)
**Residence**						
City	108 (3)	34 (1)	139 (3)	193 (6)	120 (3)	29 (2)
Town	46 (1)	45 (1)	87 (2)	141 (3)	92 (2)	30 (1)
Rural	48 (1)	70 (1)	116 (2)	151 (4)	104 (2)	19 (1)
**Income**						
1st quintile	45 (1)	55 (1)	114 (2)	117 (3)	110 (2)	24 (1)
2nd quintile	29 (1)	30 (1)	73 (2)	123 (3)	76 (2)	15 (1)
3rd quintile	45 (1)	43 (1)	78 (2)	110 (3)	68 (1)	19 (1)
4th quintile	35 (1)	11 (<1)	32 (1)	76 (2)	33 (1)	13 (1)
5th quintile	48 (1)	10 (<1)	45 (1)	59 (2)	29 (1)	7 (<1)
**Number of close people**						
None	7 (<1)	8 (<1)	13 (<1)	18 (<1)	9 (<1)	6 (<1)
1 or 2	107 (2)	85 (2)	188 (4)	240 (6)	196 (4)	53 (3)
3 or 4	64 (2)	48 (1)	112 (3)	176 (5)	86 (2)	15 (1)
5 or more	24 (1)	8 (<1)	29 (1)	51 (2)	25 (1)	4 (<1)
**Limited performance**						
No	89 (2)	41 (1)	140 (3)	252 (7)	116 (3)	24 (1)
Yes	113 (2)	108 (2)	202 (4)	233 (6)	200 (4)	54 (3)
**Self-assessed health**						
Very good	14 (<1)	5 (<1)	13 (<1)	21 (1)	8 (<1)	2 (<1)
Good	52 (1)	20 (<1)	79 (2)	123 (4)	53 (1)	11 (1)
Fair	61 (1)	43 (1)	121 (3)	209 (5)	128 (3)	24 (1)
Bad	51 (1)	55 (1)	89 (2)	107 (3)	95 (2)	29 (2)
Very bad	24 (<1)	26 (<1)	40 (1)	25 (1)	32 (1)	12 (1)
**Chronic condition**						
No	44 (1)	24 (1)	80 (2)	129 (4)	49 (1)	8 (1)
Yes	158 (3)	125 (2)	262 (5)	356 (9)	267 (6)	70 (4)
