# Supplementary material for: The prevalence and determinants of unmet healthcare needs in Bulgaria
Source: PLoS One. 2024 Oct 29;19(10):e0312475. doi: 10.1371/journal.pone.0312475 (PMC11521248; doi:10.1371/journal.pone.0312475)
Supplement: S4 Appendix — (PDF) [file pone.0312475.s004.pdf]

S4 Appendix. Variables in the statistical models

S1 Table. Explanatory variables in the final logit models

	Model:					
Variable:	1	2	3	4	5	6
Gender	x	x	x	x	x	x
Age	x	x	x	x	x	x
Employment status	x	x	x	x	x	x
Income	x	x	x	x	x	x
Household size	x	x	x	x	x	x
Education	x	x	x	x	x	x
Civil status		x	x	x	x	
Carer status	x	x	x	x	x	
Concern	x	x	x	x	x	x
Close people			x	x	x	
Residence status	x	x	x	x	x	
Self-assessed health	x	x	x	x	x	x
Presence of a depressive disorder	x	x	x	x	x	x
BMI (underweight/normal vs overweight/obese)			x	x	x	
Limited performance	x	x	x	x	x	x
Presence of chronic illness	x	x	x	x	x	x
Smoking habits	x	x	x	x	x	x
Alcohol drinking habits	x	x	x	x	x	x

S2 Table. AIC and BIC characteristics of the logit models

		Model:					
		1	2	3	4	5	6
Full model	AIC	1556	1096	2173	2468	2009	527
	BIC	1811	1352	2429	2714	2261	740
Reduced model	AIC	1549	1092	2181	2469	2014	521
	BIC	1760	1321	2373	2672	2204	681

AIC Akaike Information Criterion, BIC Bayesian Information Criterion
