# Supplementary material for: The prevalence and determinants of unmet healthcare needs in Bulgaria
Source: PLoS One. 2024 Oct 29;19(10):e0312475. doi: 10.1371/journal.pone.0312475 (PMC11521248; doi:10.1371/journal.pone.0312475)
Supplement: S5 Appendix — (PDF) [file pone.0312475.s005.pdf]

## S5 Appendix. Fully-adjusted odds ratios

| | Wait time too long | Distance or transport problems | Affordability of medical care | Affordability of dental care | Affordability of prescribed drugs | Affordability of mental healthcare |
|---|---|---|---|---|---|---|
| **Sex (ref. Male)** | | | | | | |
| Female | **1.36*** (0.96 to 1.93) | 1.03 (0.69 to 1.54) | 1.19 (0.91 to 1.55) | **1.27*** (0.98 to 1.63) | 1.04 (0.79 to 1.38) | **1.65*** (0.91 to 2.99) |
| **Age (ref. 15-29)** | | | | | | |
| 30-49 | 1.32 (0.47 to 2.74) | 2.04 (0.34 to 12.16) | 0.92 (0.43 to 1.95) | 1.56 (0.77 to 3.16) | 1.52 (0.52 to 4.41) | 0.48 (0.14 to 1.69) |
| 50-69 | 0.78 (0.33 to 1.85) | 1.25 (0.20 to 7.68) | 0.98 (0.45 to 2.16) | **2.47**** (1.19 to 5.16) | 0.99 (0.30 to 3.24) | **0.24**** (0.06 to 0.94) |
| 70+ | 0.60 (0.23 to 1.59) | 0.95 (0.14 to 6.36) | 1.33 (0.54 to 3.26) | **3.02***** (1.32 to 6.89) | 1.24 (0.35 to 4.39) | 0.36 (0.07 to 1.82) |
| **Education (ref. Primary or lower)** | | | | | | |
| Secondary | **0.53**** (0.31 to 0.89) | **0.42***** (0.25 to 0.71) | 0.70 (0.45 to 1.08) | 0.88 (0.56 to 1.38) | 0.93 (0.57 to 1.53) | 1.48 (0.58 to 3.75) |
| Tertiary | 0.62 (0.33 to 1.16) | **0.28***** (0.13 to 0.61) | 0.52 (0.30 to 0.91) | **0.44***** (0.25 to 0.77) | 0.61 (0.32 to 1.16) | **0.24**** (0.06 to 0.91) |
| **Employment (ref. Employed)** | | | | | | |
| Unemployed | 0.50 (0.26 to 1.21) | **2.11*** (0.90 to 4.98) | 1.41 (0.86 to 2.31) | 1.45 (0.92 to 2.28) | 1.21 (0.68 to 2.16) | 0.80 (0.31 to 2.06) |
| Retired | 0.79 (0.44 to 1.33) | 1.21 (0.56 to 2.61) | 0.69 (0.43 to 1.09) | 0.92 (0.63 to 1.35) | 0.93 (0.57 to 1.53) | **0.41*** (0.15 to 1.12) |
| Other inactive | **0.53*** (0.27 to 1.04) | 1.32 (0.55 to 3.18) | 0.69 (0.38 to 1.26) | 0.95 (0.55 to 1.64) | 0.86 (0.44 to 1.70) | 0.67 (0.27 to 1.67) |
| **Marital status (ref. Single)** | | | | | | |
| Married | | **2.62**** (1.23 to 5.58) | 0.80 (0.52 to 1.25) | **0.62**** (0.43 to [illegible] | 1.06 (0.63 to 1.81) | |
| Widowed | | 1.70 (0.70 to 4.13) | **0.62*** (0.36 to [illegible] | **0.48**** (0.29 to [illegible] | 0.65 (0.35 to 1.21) | |
| Divorced | | 1.88 (0.62 to 5.76) | 0.86 (0.46 to 1.60) | 0.75 (0.44 to 1.27) | 0.79 (0.39 to 1.57) | |
| **Income (ref. 1st quintile)** | | | | | | |
| 2nd quintile | 0.63 (0.36 to 1.11) | 0.71 (0.41 to 1.24) | **0.66**** (0.46 to 0.96) | 1.20 (0.85 to 1.70) | **0.71*** (0.51 to 1.05) | 0.55 (0.24 to 1.26) |
| 3rd quintile | 0.91 (0.53 to 1.55) | 1.24 (0.72 to 2.16) | **0.59***** (0.39 to [illegible] | 0.80 (0.55 to 1.15) | **0.58***** (0.38 to [illegible] | 0.60 (0.27 to 1.36) |

| | | | | | | |
|---|---|---|---|---|---|---|
| 4th quintile | 0.78 (0.42 to 1.46) | 0.51 (0.23 to 1.15) | **0.31***** (0.19 to [illegible] | **0.57***** (0.38 to [illegible] | **0.34***** (0.20 to [illegible] | 0.85 (0.35 to 2.06) |
| 5th quintile | 1.16 (0.65 to 2.07) | 0.86 (0.36 to 2.06) | **0.54**** (0.32 to [illegible] | **0.65*** (0.41 to [illegible] | **0.51**** (0.29 to [illegible] | 0.52 (0.18 to 1.52) |
| **Household size (ref. 1 person)** | | | | | | |
| 2 people | 0.73 (0.46 to 1.16) | **0.54*** (0.28 to 1.03) | 0.74 (0.47 to 1.17) | 0.95 (0.62 to 1.43) | **0.62*** (0.38 to 1.02) | 1.23 (0.60 to 2.54) |
| 3 people | 0.71 (0.40 to 1.26) | **0.38**** (0.18 to 0.79) | 1.06 (0.62 to 1.80) | 1.10 (0.68 to 1.78) | 1.07 (0.62 to 1.85) | **2.29*** (0.96 to 5.46) |
| 4+ people | 0.76 (0.44 to 1.33) | **0.55*** (0.28 то 1.08) | 0.93 (0.56 to 1.57) | 1.27 (0.80 to 2.00) | 0.90 (0.53 to 1.54) | 1.03 (0.46 to 2.30) |
| **Settlement status (ref. City)** | | | | | | |
| Town/suburb | **0.50**** (0.33 to 0.74) | 1.14 (0.69 to 1.89) | **0.54***** (0.39 to 0.74) | **0.67***** (0.51 to 0.88) | **0.61***** (0.44 to 0.84) | |
| Rural area | **0.64**** (0.44 to 0.95) | **2.68***** (1.65 to 4.34) | 1.07 (0.79 to 1.45) | 1.11 (0.84 to 1.47) | 1.07 (0.76 to 1.49) | |
| **Carer (ref. No)** | | | | | | |
| Yes | 1.35 (0.87 to 2.08) | **1.97***** (1.20 to 3.23) | 1.25 (0.88 to 1.78) | 1.19 (0.88 to 1.62) | 1.01 (0.69 to 1.48) | |
| **Concern (ref. A lot)** | | | | | | |
| Some | 0.89 (0.62 to 1.28) | 1.45 (0.93 to 2.25) | 1.22 (0.90 to 1.65) | **1.47***** (1.13 to 1.92) | **1.71***** (1.24 to 2.37) | 1.22 (0.61 to 2.43) |
| None/not sure | 0.87 (0.57 to 1.35) | **2.58***** (1.54 to 4.35) | **1.96***** (1.35 to 2.85) | **2.53***** (1.81 to 3.53) | **2.20***** (1.49 to 3.24) | **1.88*** (0.91 to 3.89) |
| **Close people (ref. None)** | | | | | | |
| 1 to 2 people | | | 1.28 (0.62 to [illegible] | 0.78 (0.40 to [illegible] | **2.02*** (0.92 to [illegible] | |
| 3 to 5 people | | | 1.26 (0.59 to 2.69) | 0.95 (0.47 to 1.91) | 1.36 (0.60 to 3.10) | |
| 6 or more | | | 1.36 (0.59 to [illegible] | 1.19 (0.56 to [illegible] | 1.93 (0.79 to [illegible] | |
| **BMI (ref. Underweight/normal)** | | | | | | |
| Overweight/ obese | | | 0.94 (0.72 to [illegible] | 0.93 (0.73 to [illegible] | 0.86 (0.65 to [illegible] | |
| **Self-assessed health (ref. Very good)** | | | | | | |
| Good | 1.56 (0.78 to [illegible] | 0.99 (0.37 to 2.73) | **2.55**** (1.26 to [illegible] | **2.37***** (1.36 to [illegible] | 1.85 (0.80 to [illegible] | 1.58 (0.30 to 8.29) |

| | | | | | | |
|---|---|---|---|---|---|---|
| Fair | **2.31*** (1.00 to 5.31) | 1.95 (0.62 to 6.07) | **3.80***** (1.79 to 8.08) | **4.72***** (2.56 to 8.72) | **3.47***** (1.47 to 8.20) | 1.99 (0.36 to 11.00) |
| Bad | **3.82**** (1.48 to 9.87) | **3.41**** (1.04 to 11.22) | **4.91***** (2.16 to 11.21) | **5.50***** (2.72 to 11.12) | **4.64***** (1.86 to 11.56) | 2.91 (0.43 to 19.72) |
| Very bad | **7.33**** (2.60 to 20.67) | **5.85***** (1.64 to 20.87) | **9.36***** (3.76 to 23.32) | **3.35***** (1.38 to 8.10) | **5.79***** (2.10 to 15.98) | 2.44 (0.31 to 18.94) |
| **Depressive disorder (ref. No)** | | | | | | |
| Yes | **2.33**** (1.47 to 3.71) | **2.55***** (1.56 to 4.14) | **1.97***** (1.35 to 2.87) | **2.37***** (1.58 to 3.56) | **2.00***** (1.30 to 3.07) | **8.10***** (3.83 to 17.16) |
| **Limited performance (ref. No)** | | | | | | |
| Yes | **1.72**** (1.08 to 2.75) | **2.12***** (1.24 to 3.63) | **1.93***** (1.34 to 2.79) | **1.54***** (1.11 to 2.13) | **1.71**** (1.18 to 2.49) | **2.55**** (1.13 to 5.74) |
| **Chronic illness (ref. No)** | | | | | | |
| Yes | 1.43 (0.85 to 2.41) | 0.65 (0.29 to 1.44) | 0.79 (0.53 to 1.17) | 0.92 (0.66 to 1.27) | 1.21 (0.75 to 1.94) | **3.19**** (1.14 to 8.89) |
| **Smoking habits (ref. Every day)** | | | | | | |
| Irregularly | 0.96 (0.46 to 1.99) | 0.79 (0.23 to 2.70) | **0.57*** (0.29 to 1.11) | 0.86 (0.53 to 1.39) | 1.34 (0.76 to 2.37) | **2.54*** (0.88 to 7.30) |
| Never | **0.61**** (0.41 to 0.91) | 1.24 (0.74 to 2.07) | **0.57***** (0.41 to 0.79) | **0.54***** (0.41 to 0.72) | 0.76 (0.53 to 1.10) | 0.71 (0.37 to 1.38) |
| **Alcohol drinking (ref. Regularly)** | | | | | | |
| Irregularly | **1.48*** (0.97 to 2.26) | 0.85 (0.51 to 1.39) | 0.82 (0.59 to 1.13) | 0.91 (0.68 to 1.21) | 1.31 (0.93 to 1.86) | 0.65 (0.30 to 1.39) |
| Never | 0.90 (0.55 to 1.48) | 0.80 (0.46 to 1.42) | 0.76 (0.52 to 1.09) | **0.65***** (0.47 to 0.90) | 1.11 (0.75 to 1.64) | 0.63 (0.29 to 1.35) |
| **Cons.** | 0.07 | 0.01 | 0.09 | 0.06 | 0.02 | 0.01 |

*Fully-adjusted odds ratios with associated 95% confidence intervals. *significant at 10%, **significant at 5%, ***significant at 1%.*

**Table S9. Conditional expected probabilities of reporting unmet needs for selected parameters**

| | Wait time was too long | Distance or transport problems | Could not afford medical care | Could not afford dental care | Could not afford pre-scribed drugs | Could not afford mental healthcare |
|---|---|---|---|---|---|---|
| **Limiter performance** | | | | | | |
| No | 3.7 | 1.9 | 5.6 | 11.4 | 5.2 | 2.9 |
| Yes | 6.1 | 3.8 | 9.9 | 15.8 | 8.3 | 6.2 |

| | | | | | | |
|---|---|---|---|---|---|---|
| **Employment** | | | | | | |
| Employed | 5.5 | 2.3 | 8.4 | 12.9 | 6.9 | 6.5 |
| Unemployed | 3.3 | 4.4 | 11.1 | 16.8 | 8.1 | 5.5 |
| Retired | 4.4 | 2.8 | 6.1 | 12.1 | 6.5 | 3.3 |
| Other | 3.1 | 3.0 | 6.2 | 12.4 | 6.1 | 4.9 |
| **Concern** | | | | | | |
| A lot | 4.9 | 1.9 | 5.9 | 9.7 | 4.6 | 3.6 |
| Some | 4.5 | 2.7 | 7.1 | 13.1 | 7.4 | 4.3 |
| None | 4.4 | 4.4 | 10.5 | 19.4 | 9.1 | 6.0 |
| **Depressive disorder** | | | | | | |
| No | 4.2 | 2.4 | 6.7 | 12.1 | 6.1 | 2.5 |
| Yes | 8.9 | 5.3 | 11.8 | 22.4 | 10.9 | 14.5 |
| **Chronic condition** | | | | | | |
| No | 3.7 | 3.7 | 8.4 | 13.4 | 5.9 | 2.1 |
| Yes | 5.2 | 2.6 | 6.9 | 12.6 | 7.0 | 5.5 |
