# Supplementary material for: The prevalence and determinants of unmet healthcare needs in Bulgaria
Source: PLoS One. 2024 Oct 29;19(10):e0312475. doi: 10.1371/journal.pone.0312475 (PMC11521248; doi:10.1371/journal.pone.0312475)
Supplement: S6 Appendix — (PDF) [file pone.0312475.s006.pdf]

S6 Appendix. Fully adjusted coefficients with a latent variable in each model

	Wait time				Distance/transportation				Medical			
	Coefficient	p	Lower 95CI	Upper 95CI	Coefficient	p	Lower 95CI	Upper 95CI	Coefficient	p	Lower 95CI	Upper 95CI
**Sex (ref. Male)**												
Female	0.37	0.11	-0.09	0.82	-0.02	0.95	-0.55	0.52	0.73	0.09	-0.12	1.59
**Age (ref. 15-29)**												
30-49	0.14	0.80	-0.93	1.20	0.73	0.43	-1.06	2.52	0.08	0.94	-2.01	2.18
50-69	-0.26	0.68	-1.46	0.95	0.48	0.63	-1.46	2.42	0.87	0.46	-1.44	3.19
70+	-0.50	0.47	-1.85	0.85	0.28	0.79	-1.79	2.35	1.92	0.15	-0.68	4.52
**Education (ref. Primary)**												
Second.	-0.98	0.00	-1.64	-0.32	-1.21	0.00	-1.83	-0.58	-1.30	0.03	-2.43	-0.16
Tertiary	-0.74	0.07	-1.55	0.07	-1.79	0.00	-2.81	-0.76	-2.10	0.01	-3.73	-0.47
**Employment (ref. Employed)**												
Unemp.	-0.63	0.21	-1.60	0.34	0.87	0.07	-0.08	1.82	1.64	0.03	0.14	3.13
Retired	-0.17	0.63	-0.86	0.52	0.27	0.56	-0.63	1.16	-0.85	0.23	-2.25	0.54
Other	-0.74	0.11	-1.64	0.16	0.23	0.66	-0.80	1.26	-0.92	0.27	-2.56	0.72
**Income (ref. 1st quintile)**												
2nd	-0.74	0.03	-1.39	-0.09	-0.46	0.16	-1.09	0.17	-1.08	0.04	-2.10	-0.05
3rd	-0.08	0.81	-0.70	0.55	0.17	0.60	-0.47	0.81	-1.10	0.05	-2.21	0.01
4th	-0.55	0.13	-1.28	0.17	-1.25	0.01	-2.18	-0.31	-3.83	0.00	-5.54	-2.13
5th	-0.04	0.92	-0.81	0.74	-0.48	0.36	-1.51	0.55	-1.96	0.02	-3.63	-0.30
**Household size (ref. 1 person)**												
2	-0.56	0.09	-1.22	0.09	-0.77	0.04	-1.52	-0.02	-0.97	0.10	-2.13	0.20
3	-0.52	0.17	-1.27	0.22	-0.93	0.05	-1.86	0.00	0.19	0.79	-1.17	1.55
4+	-0.36	0.32	-1.07	0.34	-0.57	0.17	-1.39	0.25	-0.33	0.62	-1.61	0.96
**Civil status (ref. Single)**												
Married	-0.15	0.67	-0.82	0.53	0.58	0.22	-0.34	1.51	-1.30	0.06	-2.63	0.03
Widowed	-0.59	0.17	-1.44	0.26	0.19	0.73	-0.89	1.26	-1.75	0.03	-3.33	-0.18
Divorced	-0.17	0.71	-1.10	0.75	0.44	0.49	-0.81	1.70	-0.61	0.49	-2.33	1.11
**Carer status (ref. No)**												
Yes	0.26	0.34	-0.28	0.81	0.92	0.00	0.30	1.54	1.14	0.03	0.09	2.18
**Concern (ref. A lot)**												
Some	-0.03	0.90	-0.47	0.41	0.57	0.05	0.01	1.13	0.82	0.07	-0.07	1.71
None	-0.13	0.66	-0.72	0.45	0.82	0.01	0.17	1.47	1.72	0.00	0.62	2.82
**Close people (ref. None)**												
1or2	-0.30	0.63	-1.50	0.91	-0.14	0.83	-1.37	1.10	0.81	0.47	-1.40	3.01
3to5	-0.51	0.42	-1.76	0.73	-0.09	0.89	-1.38	1.20	0.89	0.45	-1.41	3.19
6+	-0.24	0.73	-1.60	1.12	-0.27	0.74	-1.82	1.29	0.90	0.49	-1.68	3.49
**Residence (ref. City)**												

Town	-0.86	0.00	-1.36	-0.36	0.19	0.54	-0.41	0.79	-1.85	0.00	-2.86	-0.83
Rural	-0.53	0.04	-1.05	-0.02	1.05	0.00	0.47	1.64	0.09	0.84	-0.80	0.99
**Health ststus (ref. Very good)**												
Good	0.67	0.12	-0.17	1.51	0.28	0.67	-1.01	1.57	3.33	0.00	1.23	5.42
Fair	1.19	0.02	0.23	2.16	0.84	0.24	-0.57	2.25	4.85	0.00	2.46	7.23
Bad	1.79	0.00	0.70	2.89	1.58	0.04	0.07	3.10	5.62	0.00	2.99	8.25
Very bad	2.45	0.00	1.14	3.75	2.18	0.01	0.50	3.86	7.15	0.00	4.10	10.21
**Depression (ref. No)**												
Yes	1.18	0.00	0.58	1.78	1.20	0.00	0.58	1.82	2.21	0.00	1.09	3.34
**BMI (ref. Normal)**												
Obese	0.11	0.59	-0.30	0.52	-0.29	0.22	-0.76	0.18	-0.08	0.83	-0.81	0.65
**Disability (ref. No)**												
Yes	0.55	0.04	0.03	1.08	1.05	0.00	0.38	1.71	1.86	0.00	0.76	2.95
**Chronic illness (ref. No)**												
Yes	0.57	0.06	-0.03	1.17	-0.32	0.43	-1.11	0.47	-0.68	0.25	-1.83	0.48
**Smoking status (ref. Yes, often)**												
Yes, sometim es	-0.19	0.68	-1.11	0.72	-0.08	0.91	-1.41	1.25	-1.69	0.07	-3.49	0.10
No	-0.64	0.01	-1.15	-0.14	0.34	0.34	-0.35	1.03	-1.84	0.00	-2.89	-0.79
**Alcohol drinking (ref. Often)**												
Irregular	0.51	0.06	-0.02	1.04	-0.16	0.63	-0.82	0.49	-0.12	0.81	-1.13	0.89
Never	-0.16	0.60	-0.77	0.44	-0.38	0.28	-1.06	0.30	-0.60	0.28	-1.69	0.49
**U**	2.13	0.00	1.77	2.48	2.03	0.00	1.65	2.40	5.96	0.00	4.45	7.47
Cons	-3.59	0.00	-5.50	-1.68	-6.06	0.00	-8.50	-3.62	-9.90	0.00	-14.2 1	-5.60

	**Dental**				**Drug**				**Mental**			
	Coeffici ent	p	Lower 95CI	Upper 95CI	Coeffici ent	p	Lower 95CI	Upper 95CI	Coeffici ent	p	Lower 95CI	Upper 95CI
**Sex**												
Female	0.20	0.25	-0.14	0.54	0.14	0.69	-0.54	0.82	0.65	0.20	-0.35	1.65
**Age**												
30-49	0.72	0.10	-0.15	1.59	0.51	0.59	-1.36	2.38	-1.38	0.21	-3.56	0.79
50-69	1.29	0.01	0.34	2.24	0.31	0.77	-1.73	2.36	-2.90	0.02	-5.39	-0.41
70+	1.40	0.01	0.34	2.46	1.07	0.35	-1.17	3.31	-2.33	0.12	-5.23	0.57
**Education**												
2nd	-0.33	0.24	-0.88	0.22	-0.11	0.82	-1.06	0.84	-0.85	0.23	-2.25	0.55
3rt	-1.25	0.00	-1.96	-0.54	-0.96	0.16	-2.29	0.37	-3.51	0.00	-5.93	-1.09
**Employment**												
Unem- ployed	0.47	0.12	-0.13	1.07	0.71	0.28	-0.58	2.00	0.14	0.89	-1.88	2.17

Retired	-0.09	0.73	-0.62	0.43	-0.16	0.79	-1.30	0.99	-0.54	0.54	-2.26	1.17
Other	-0.22	0.53	-0.91	0.47	-0.90	0.20	-2.27	0.48	-0.39	0.65	-2.07	1.28
**Income**												
2nd	0.22	0.35	-0.24	0.67	-0.95	0.02	-1.75	-0.14	-0.80	0.20	-2.02	0.41
3rd	-0.28	0.25	-0.76	0.20	-1.48	0.00	-2.40	-0.56	-1.20	0.08	-2.56	0.15
4th	-0.70	0.01	-1.25	-0.14	-2.87	0.00	-4.12	-1.62	-1.68	0.05	-3.33	-0.04
5th	-0.62	0.05	-1.25	0.00	-2.24	0.00	-3.61	-0.87	-1.77	0.08	-3.74	0.19
**Household size**												
2	-0.13	0.61	-0.64	0.37	-1.09	0.03	-2.05	-0.12	0.00	1.00	-1.40	1.41
3	0.29	0.33	-0.29	0.87	0.14	0.81	-0.99	1.27	1.36	0.11	-0.29	3.01
4	0.27	0.34	-0.28	0.82	-0.29	0.60	-1.35	0.78	-0.39	0.63	-2.00	1.21
**Civil status**												
Married	-0.61	0.02	-1.14	-0.09	-0.86	0.12	-1.95	0.22	0.13	0.86	-1.32	1.59
Widowed	-0.87	0.01	-1.52	-0.22	-1.78	0.01	-3.08	-0.48	-0.54	0.58	-2.45	1.37
Divorced	-0.35	0.33	-1.05	0.35	-0.93	0.22	-2.42	0.55	0.69	0.48	-1.23	2.61
**Carer status**												
Yes	0.44	0.04	0.02	0.86	0.24	0.59	-0.63	1.10	0.14	0.83	-1.12	1.41
**Concern**												
Some	0.56	0.00	0.20	0.92	1.45	0.00	0.68	2.23	0.62	0.26	-0.47	1.71
None	1.19	0.00	0.75	1.63	1.85	0.00	0.94	2.76	1.36	0.03	0.10	2.61
**Close people**												
1or2	-0.63	0.22	-1.62	0.37	1.51	0.13	-0.42	3.44	-1.87	0.06	-3.84	0.10
3to5	-0.47	0.37	-1.48	0.55	0.99	0.33	-1.00	2.98	-1.70	0.12	-3.85	0.44
6+	-0.20	0.73	-1.30	0.90	1.35	0.24	-0.88	3.57	-1.27	0.35	-3.94	1.39
**Residence**												
Town	-0.56	0.00	-0.93	-0.18	-1.15	0.00	-1.91	-0.39	0.20	0.69	-0.79	1.20
Rural	-0.01	0.98	-0.39	0.38	-0.12	0.74	-0.86	0.61	-0.19	0.75	-1.34	0.97
**Health status**												
Good	1.03	0.00	0.34	1.73	1.91	0.04	0.13	3.70	0.58	0.64	-1.83	2.98
Fair	2.15	0.00	1.38	2.92	3.94	0.00	1.92	5.96	1.46	0.27	-1.14	4.07
Bad	2.30	0.00	1.42	3.18	4.49	0.00	2.32	6.65	1.95	0.17	-0.82	4.72
Very bad	1.53	0.01	0.42	2.64	4.56	0.00	2.15	6.97	1.28	0.41	-1.76	4.33
**Depression**												
Yes	1.34	0.00	0.81	1.87	1.88	0.00	0.99	2.77	3.94	0.00	2.59	5.30
**BMI**												
Obese	-0.09	0.60	-0.40	0.23	-0.24	0.43	-0.85	0.36	0.36	0.42	-0.52	1.24
**Disability**												
Yes	0.45	0.03	0.05	0.85	1.27	0.00	0.47	2.08	1.34	0.03	0.13	2.54
**Chronic illness**												
Yes	-0.02	0.93	-0.45	0.41	0.30	0.55	-0.68	1.29	1.38	0.06	-0.07	2.83

**Smoking status**												
Yes, sometimes	0.18	0.58	-0.46	0.82	0.88	0.18	-0.41	2.17	2.11	0.02	0.36	3.86
None	-0.74	0.00	-1.13	-0.35	-0.82	0.06	-1.66	0.03	-0.95	0.14	-2.22	0.31
**Alcohol drinking**												
Irregular	-0.09	0.63	-0.48	0.29	0.97	0.03	0.11	1.82	-0.76	0.24	-2.04	0.52
Never	-0.50	0.03	-0.94	-0.06	0.42	0.37	-0.49	1.32	-0.52	0.45	-1.85	0.81
**U**	2.00	0.00	1.71	2.30	4.78	0.00	3.76	5.80	3.41	0.00	2.32	4.50
Cons	-3.90	0.00	-5.48	-2.32	-10.89	0.00	-14.63	-7.15	-4.83	0.02	-8.94	-0.72
