# Supplementary figures and images for: The prevalence and determinants of unmet healthcare needs in Bulgaria
Source: PLoS One. 2024 Oct 29;19(10):e0312475. doi: 10.1371/journal.pone.0312475 (PMC11521248; doi:10.1371/journal.pone.0312475)

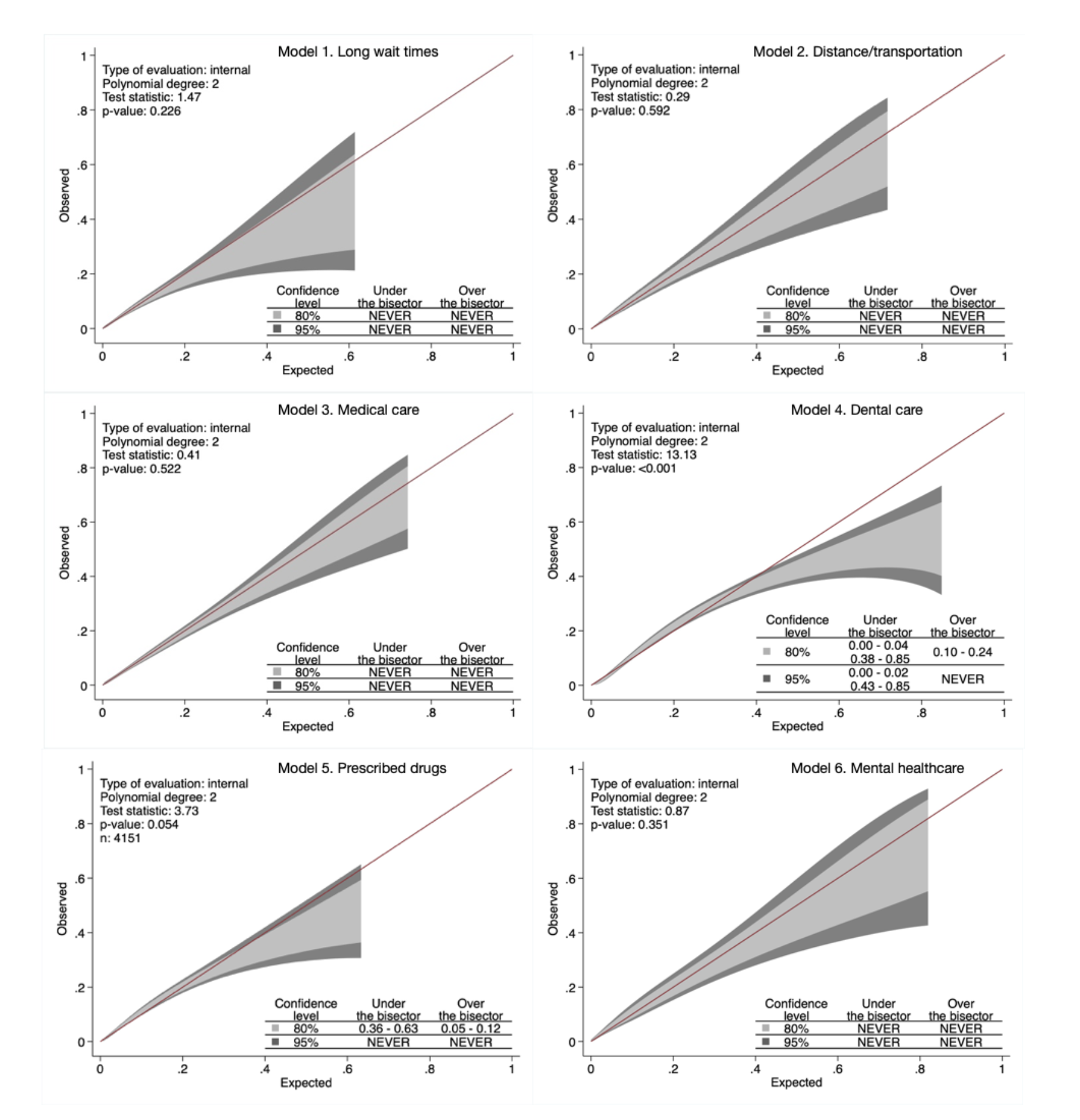

Supplement: S1 Fig — (PNG) [file pone.0312475.s007.png]

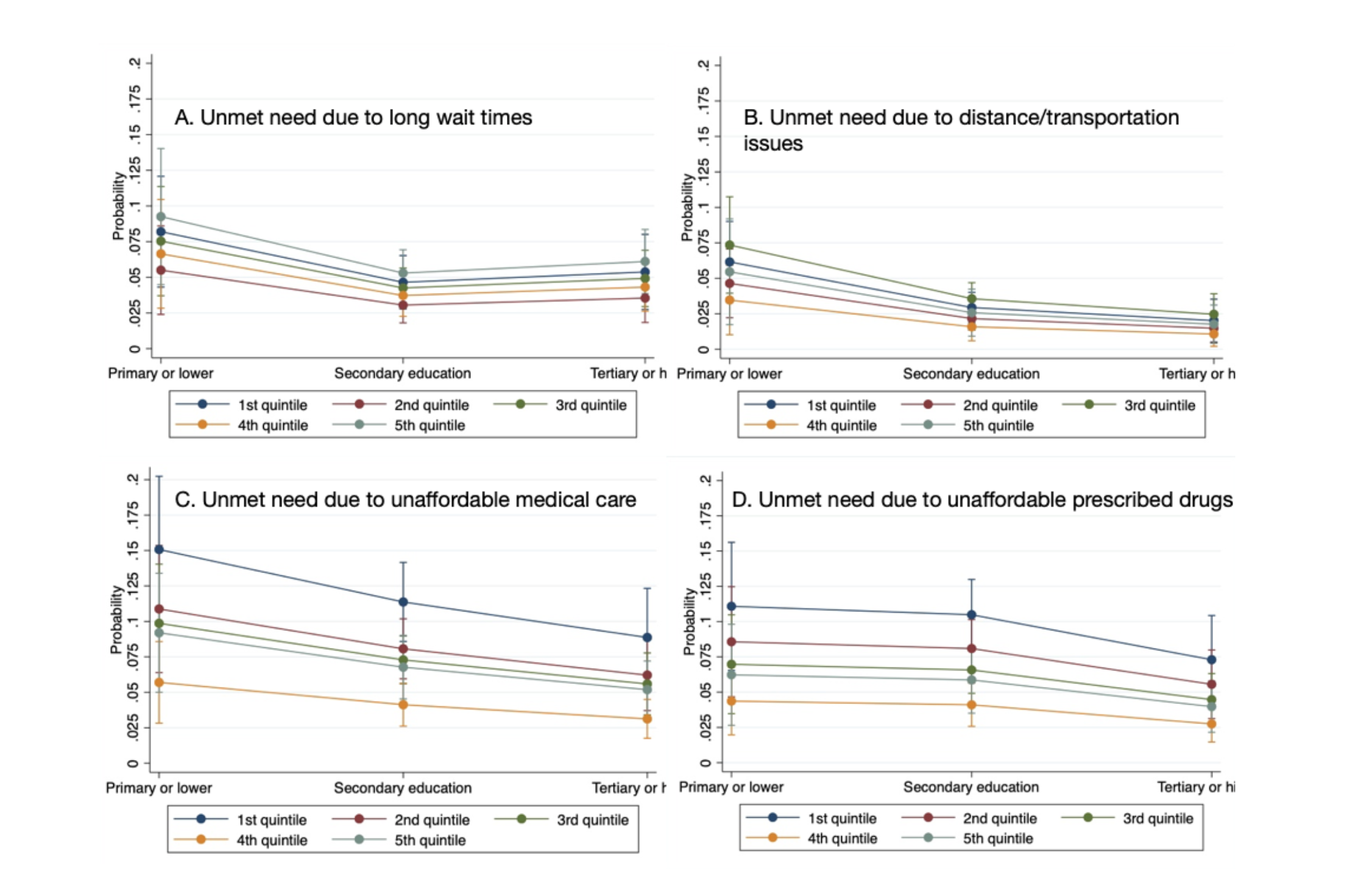

Supplement: S2 Fig — (PNG) [file pone.0312475.s008.png]

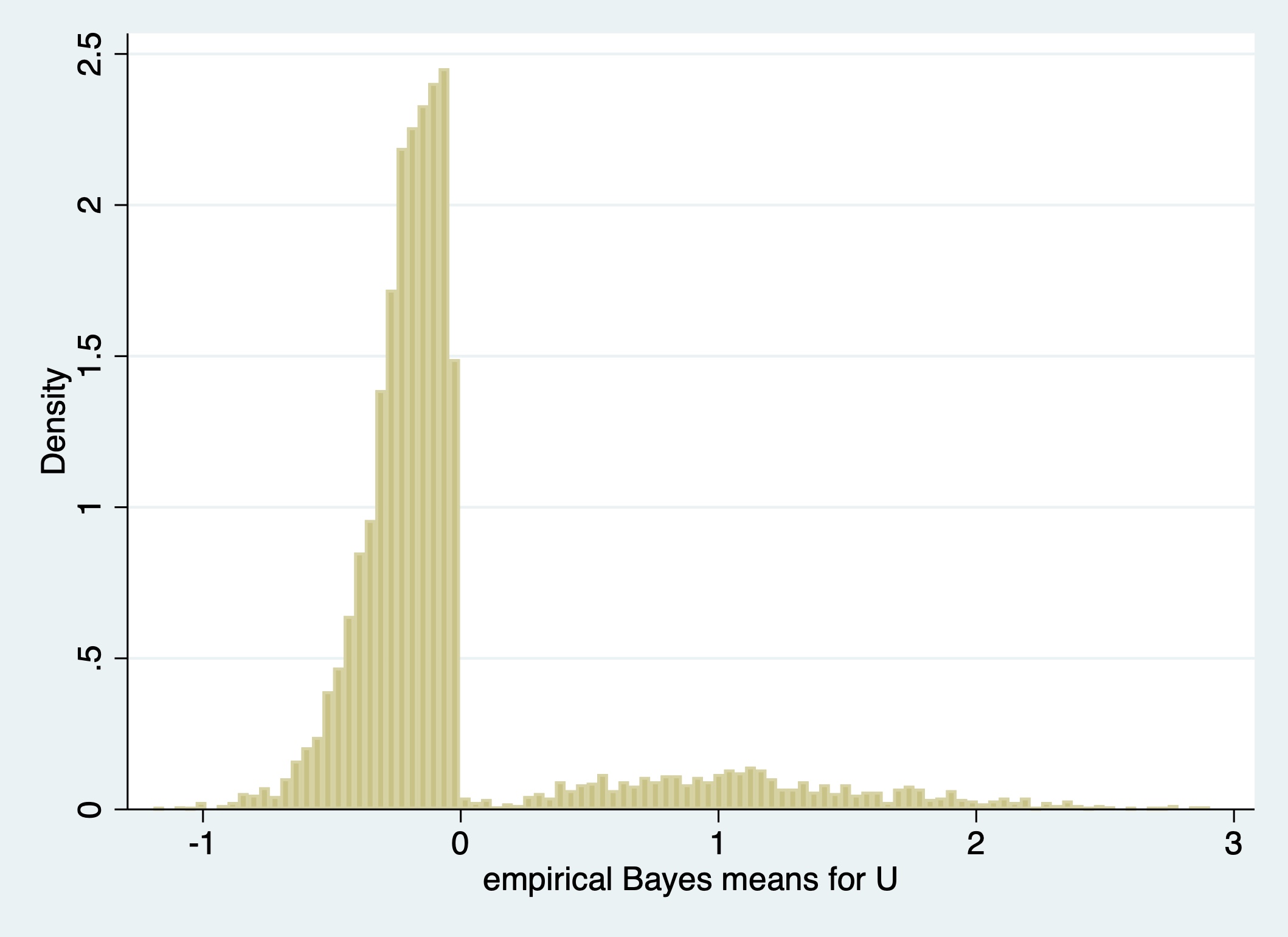

Supplement: S3 Fig — (JPG) [file pone.0312475.s009.jpg]
